# Associations of maternal dietary inflammatory potential and quality with offspring birth outcomes: An individual participant data pooled analysis of 7 European cohorts in the ALPHABET consortium

**DOI:** 10.1371/journal.pmed.1003491

**Published:** 2021-01-21

**Authors:** Ling-Wei Chen, Adrien M. Aubert, Nitin Shivappa, Jonathan Y. Bernard, Sara M. Mensink-Bout, Aisling A. Geraghty, John Mehegan, Matthew Suderman, Kinga Polanska, Wojciech Hanke, Elzbieta Trafalska, Caroline L. Relton, Sarah R. Crozier, Nicholas C. Harvey, Cyrus Cooper, Liesbeth Duijts, Barbara Heude, James R. Hébert, Fionnuala M. McAuliffe, Cecily C. Kelleher, Catherine M. Phillips

**Affiliations:** 1 HRB Centre for Health and Diet Research, School of Public Health, Physiotherapy, and Sports Science, University College Dublin, Dublin, Republic of Ireland; 2 Université de Paris, Centre for Research in Epidemiology and StatisticS (CRESS), Inserm, Inrae, Paris, France; 3 Cancer Prevention and Control Program, Arnold School of Public Health, University of South Carolina, Columbia, South Carolina, United States of America; 4 Connecting Health Innovations, LLC, Columbia, South Carolina, United States of America; 5 Singapore Institute for Clinical Sciences (SICS), Agency for Science, Technology and Research (A*STAR), Singapore, Singapore; 6 The Generation R Study Group, Erasmus MC, University Medical Center, Rotterdam, the Netherlands; 7 Department of Pediatrics, Division of Respiratory Medicine and Allergology, Erasmus MC, University Medical Center Rotterdam, Rotterdam, the Netherlands; 8 UCD Perinatal Research Centre, School of Medicine, University College Dublin, National Maternity Hospital, Dublin, Republic of Ireland; 9 MRC Integrative Epidemiology Unit, University of Bristol, Bristol, United Kingdom; 10 Nofer Institute of Occupational Medicine, Lodz, Poland; 11 Department of Hygiene and Epidemiology, Medical University of Lodz, Lodz, Poland; 12 MRC Lifecourse Epidemiology Unit (University of Southampton) Southampton General Hospital, Southampton, United Kingdom; 13 Department of Pediatrics, Division of Neonatology, Erasmus MC, University Medical Center, Rotterdam, the Netherlands; University of Edinburgh, UNITED KINGDOM

## Abstract

**Background:**

Adverse birth outcomes are major causes of morbidity and mortality during childhood and associate with a higher risk of noncommunicable diseases in adult life. Maternal periconception and antenatal nutrition, mostly focusing on single nutrients or foods, has been shown to influence infant birth outcomes. However, evidence on whole diet that considers complex nutrient and food interaction is rare and conflicting. We aim to elucidate the influence of whole-diet maternal dietary inflammatory potential and quality during periconceptional and antenatal periods on birth outcomes.

**Methods and findings:**

We harmonized and pooled individual participant data (IPD) from up to 24,861 mother–child pairs in 7 European mother–offspring cohorts [cohort name, country (recruitment dates): ALSPAC, UK (1 April 1991 to 31 December 1992); EDEN, France (27 January 2003 to 6 March 2006); Generation R, the Netherlands (1 April 2002 to 31 January 2006); Lifeways, Ireland (2 October 2001 to 4 April 2003); REPRO_PL, Poland (18 September 2007 to 16 December 2011); ROLO, Ireland (1 January 2007 to 1 January 2011); SWS, United Kingdom (6 April 1998 to 17 December 2002)]. Maternal diets were assessed preconceptionally (*n* = 2 cohorts) and antenatally (*n* = 7 cohorts). Maternal dietary inflammatory potential and quality were ranked using the energy-adjusted Dietary Inflammatory Index (E-DII) and Dietary Approaches to Stop Hypertension (DASH) index, respectively. Primary outcomes were birth weight and gestational age at birth. Adverse birth outcomes, i.e., low birth weight (LBW), macrosomia, small-for-gestational-age (SGA), large-for-gestational-age (LGA), preterm and postterm births were defined according to standard clinical cutoffs. Associations of maternal E-DII and DASH scores with infant birth outcomes were assessed using cohort-specific multivariable regression analyses (adjusted for confounders including maternal education, ethnicity, prepregnancy body mass index (BMI), maternal height, parity, cigarettes smoking, and alcohol consumption), with subsequent random-effects meta-analyses.

Overall, the study mothers had a mean ± SD age of 29.5 ± 4.9 y at delivery and a mean BMI of 23.3 ± 4.2 kg/m^2^. Higher pregnancy DASH score (higher dietary quality) was associated with higher birth weight [β(95% CI) = 18.5(5.7, 31.3) g per 1-SD higher DASH score; *P* value = 0.005] and head circumference [0.03(0.01, 0.06) cm; *P* value = 0.004], longer birth length [0.05(0.01, 0.10) cm; *P* value = 0.010], and lower risk of delivering LBW [odds ratio (OR) (95% CI) = 0.89(0.82, 0.95); *P* value = 0.001] and SGA [0.87(0.82, 0.94); *P* value < 0.001] infants. Higher maternal prepregnancy E-DII score (more pro-inflammatory diet) was associated with lower birth weight [β(95% CI) = −18.7(−34.8, −2.6) g per 1-SD higher E-DII score; *P* value = 0.023] and shorter birth length [−0.07(−0.14, −0.01) cm; *P* value = 0.031], whereas higher pregnancy E-DII score was associated with a shorter birth length [−0.06(−0.10, −0.01) cm; *P* value = 0.026] and higher risk of SGA [OR(95% CI) = 1.18(1.11, 1.26); *P* value < 0.001]. In male, but not female, infants higher maternal prepregnancy E-DII was associated with lower birth weight and head circumference, shorter birth length, and higher risk of SGA (*P*-for-sex-interaction = 0.029, 0.059, 0.104, and 0.075, respectively). No consistent associations were observed for maternal E-DII and DASH scores with gestational age, preterm and postterm birth, or macrosomia and LGA. Limitations of this study were that self-reported dietary data might have increased nondifferential measurement error and that causality cannot be claimed definitely with observational design.

**Conclusions:**

In this cohort study, we observed that maternal diet that is of low quality and high inflammatory potential is associated with lower offspring birth size and higher risk of offspring being born SGA in this multicenter meta-analysis using harmonized IPD. Improving overall maternal dietary pattern based on predefined criteria may optimize fetal growth and avert substantial healthcare burden associated with adverse birth outcomes.

## Abstract 2

In this cohort analysis, Ling-Wei Chen and colleagues explore associations of maternal dietary patterns with offspring birth outcomes.

## Author summary

**Why was this study done?:**

- Adverse birth outcomes are associated with higher morbidity and mortality during childhood and a higher risk of noncommunicable diseases in adult life.
- The Developmental Origins of Health and Diseases (DOHaD) theory posits that maternal periconceptional and intrauterine nutrition can alter the health trajectory of the offspring.
- Although individual maternal dietary factors have been studied widely, evidence on the impact of whole-diet maternal dietary inflammatory potential and quality on birth outcomes is scarce and conflicting.

**What did the researchers do and find?:**

- We investigated whether maternal prepregnancy and antenatal dietary quality and inflammatory potential are associated with birth outcomes in a consortium of 7 European cohorts in 5 countries using harmonized individual participant data from up to 24,861 mother–child pairs.
- After adjusting for confounders, we found that a low-quality and pro-inflammatory maternal diet during pregnancy is significantly associated with lower offspring birth weight and higher risk of offspring being born small-for-gestational-age (SGA).
- In male, but not female, infants higher maternal prepregnancy energy-adjusted Dietary Inflammatory Index (E-DII) score was associated with lower birth weight and head circumference, shorter birth length, and higher risk of SGA.

**What do these findings mean?:**

- Improving overall maternal dietary quality and reducing dietary inflammatory potential may optimize fetal growth and avert substantial healthcare burden associated with adverse birth outcomes.
- Policies to ensure availability of affordable healthy foods and programmatic efforts to inform and support women of reproductive age, such as raising awareness of the importance of maternal diet and prenatal and antenatal counseling would help women achieve a healthier diet.

## Introduction

Adverse birth outcomes including low birth weight (LBW), macrosomia, and preterm or postterm birth are associated with higher morbidity and mortality during childhood and higher risk of noncommunicable diseases in adult life [1–4]. Globally, it has been estimated that LBW and preterm birth occur in 15% to 20% and 5% to 18% of all livebirths, respectively [5,6]. Thus, identifying modifiable risk factors of adverse birth outcomes is paramount for improving both child and adult health outcomes.

The Developmental Origins of Health and Diseases (DOHaD) theory posits that maternal periconceptional and intrauterine nutrition can alter the health trajectory of the offspring. Several lines of evidence from animal studies [7], human famine studies [8,9], and recent human studies with milder nutritional challenge [10,11] support this theory. Nonetheless, human diet in free-living conditions is complex with high correlations and interactions among nutrients and foods [12]. Furthermore, the effect of individual nutrients and foods on health may be too small to be detected in studies with average sample size [13]. By examining diet as a whole and measuring several aspects of dietary intake against dietary recommendations, diet quality indices offer a holistic view of diet and could be more effective for public health messaging [14]. Pregnancy is also known to alter maternal immune and inflammatory milieu [15], and imbalance in inflammatory cytokines is in turn associated with pregnancy complications and adverse birth outcomes [16,17]. Because diet is a major modifiable factor of inflammation [18,19], one of the potential strategies in optimizing birth outcomes could be through reducing dietary inflammation.

Despite the importance of dietary quality and inflammatory potential, limited studies have investigated their relationships with birth outcomes, with inconsistent findings reported [20,21]. Although there have been recent attempts to synthesize evidence surrounding maternal dietary patterns and birth outcomes using aggregate data meta-analysis, notable heterogeneity of dietary pattern derivation (data-driven or predefined criteria), adjustment and analysis strategy, and what constitutes a “healthy pattern” impedes conclusive inference [20,21]. To this end, conducting an individual participant data meta-analysis implementing a harmonized data and analysis approach is a strategy which is likely to reduce clinical heterogeneity and yield more robust evidence. We thus investigate whether maternal prepregnancy and antenatal dietary quality and inflammatory potential are associated with birth outcomes in a consortium of 7 European cohorts in 5 countries using harmonized individual participant data.

## Methods

### Study population

This study involves 7 mother–offspring cohort studies across 5 European countries within the ALPHABET consortium, formed in 2017 with an overarching aim to investigate the complex interplay between maternal dietary environment, epigenetics, and a range of child health outcomes. These cohorts/longitudinal follow-up from a randomized controlled trial include the Lifeways Cross-Generation Cohort Study (Lifeways; recruitment from 2 October 2001 to 4 April 2003) and the Randomised cOntrol trial of LOw glycaemic index diet during pregnancy study (ROLO; recruitment from 1 January 2007 to 1 January 2011) in Ireland, the study on the pre- and early postnatal determinants of child health and development (EDEN; recruitment from 27 January 2003 to 6 March 2006) in France, the Avon Longitudinal Study of Parents and Children (ALSPAC; recruitment from 1 April 1991 to 31 December 1992) and the Southampton Women’s Survey (SWS; recruitment from 6 April 1998 to 17 Dec 2002) in the United Kingdom, the Polish Mother and Child Cohort (REPRO_PL; recruitment from 18 September 2007 to 16 December 2011) in Poland, and The Generation R Study (Generation R; recruitment of pregnant women with an expected delivery date between 1 April 2002 and 31 January 2006) in the Netherlands. The study design for each cohort has been described in detail elsewhere [22–30]. Following the signing of consortium and data transfer agreements, anonymized individual participant data were transferred to University College Dublin, Ireland, for analysis. The characteristics of each study and numbers of participants included for the current analysis are summarized in S1 Table. We followed the planning and analysis approach laid out in our project protocol for funding application (see S2 Text for the extracted part for this work package; only part of the plan is relevant as our work package consists of several substudies). This study is reported according to the Preferred Reporting Items for Systematic Reviews and Meta-Analyses of Individual Participants Data (PRISMA-IPD) guideline (S1 PRISMA Checklist).

### Ethics statement

All studies have been approved by the respective local ethical review committees (listed in S2 Table), and written informed consent was obtained from all mothers.

### Exposure

#### Maternal dietary assessment

Prepregnancy or antenatal dietary intakes of the study mothers were assessed using validated (except ALSPAC) food frequency questionnaire (FFQ), which have been described in detail elsewhere [31–38]. Intake for a comprehensive list of site-specific foods (mean in ALPHABET: 137 food items) were declared on a frequency scale ranging from 5 to 9 response categories. For comparability across the consortium, the reported frequency was standardized as frequency of consumption per day (e.g., “once a week” was converted to daily frequency using the formula 1/7). In ALPHABET, prepregnancy maternal diet was available in 2 studies (SWS and EDEN), while pregnancy maternal diet was assessed in all studies. Pregnancy diet was further classified based on period of assessment: early pregnancy (first/early second trimester; *n* = 5 cohorts) and late pregnancy (third trimester; *n* = 3 cohorts) (maternal diet was assessed during both early and late pregnancy in SWS; both were included and the average was taken to reflect overall pregnancy exposure).

#### Derivation of maternal dietary inflammatory potential score

Maternal dietary inflammatory potential was scored using the energy-adjusted (using density method) Dietary Inflammatory Index (E-DII), a well-validated literature-derived score derived from the Dietary Inflammatory Index (DII) of which development has been described in detail elsewhere [39]. Briefly, dietary information for each mother was converted to amount per 1,000-kcal values and then linked to a regionally representative database, which provides an overall estimate of mean and standard deviation of energy-standardized intakes for each of the dietary parameters (i.e., nutrients, foods, and other food components), which also were adjusted for energy using the density method. By subtracting the mean of the energy-adjusted regionally representative database from the participants-reported amount and dividing this value by the parameter’s representative standard deviation, z-scores for each dietary parameter were derived. These z-scores were converted to cumulative proportions (i.e., with values ranging from 0 to 1) and then centered by doubling and subtracting 1. The resulting value was then multiplied by the corresponding food parameter effect score (derived from a comprehensive literature review of 1,943 peer-reviewed articles). These food parameter-specific E-DII scores were then summed to yield the overall E-DII score. A higher E-DII score indicates a more pro-inflammatory diet. The E-DII score in ALPHABET was generated from 24 to 28 (out of 44 possible) dietary parameters in all cohorts except for Generation R, which has 20 dietary parameters (S3 Table).

#### Derivation of maternal dietary quality score

Dietary quality was assessed by degree of adherence to the Dietary Approaches to Stop Hypertension (DASH) diet. The harmonization and moderation process for DASH score generation within the ALPHABET consortium has been described elsewhere [40]. In ALPHABET, we used 48.1% to 79.1% of the total FFQ food items (excluding alcohol) for creating the DASH score (ALPHABET’s mean = 57.8%). Most food components comprised a significant number of food items: at least 5 food items (S3 Table). The DASH score in ALPHABET was generated based mainly on the index proposed by Fung and colleagues [41], which ranks an individual’s diet based on population quintile ranking. Compared with other methods based on whether one meets recommended servings of foods [42], we deemed Fung’s and colleagues’ approach more suitable for our data derived from FFQ, which aims to rank participants according to their intakes rather than for absolute estimation of food intakes. The final ALPHABET DASH score is composed of 8 components. Participants in the highest quintile received a score of 5 for food components with higher intake recommended (fruits, vegetables excluding potatoes, total grains, non-full-fat dairy products, and nuts/seeds/legumes), while those in the lowest quintile received a score of 1. Reverse scoring was applied to food components with moderation recommended (red and processed meats, sugar-sweetened beverages/sweets/added sugars, and sodium). A higher DASH score reflects a higher dietary quality.

### Outcomes

Primary outcomes were birth weight and gestational age, both modeled continuously and categorized based on clinical cutoffs as follows: (1) low birth weight (LBW, i.e., birth weight <2,500 g); (2) macrosomia (i.e., birth weight >4,000 g); (3) preterm birth (delivery at <37 completed weeks of gestation); and (4) postterm birth (delivery at ≥42 completed weeks of gestation) [4,43,44]. Furthermore, small-for-gestational-age (SGA) and large-for-gestational-age (LGA) were defined as having sex-and-gestational-age-specific birth weight <10th and >90th percentiles, respectively, based on the INTERGROWTH-21 birthweight-for-gestational-age reference [45,46]. Because the included range of gestational age in the reference is 24 to 42 completed gestational weeks, infants with gestational age outside this range were excluded from SGA and LGA calculation. The proportions of infants with adverse birth outcomes are shown in S4 Table.

Secondary outcomes were birth length and head circumference measured at birth. In some cohorts (EDEN, ROLO, and SWS), abdominal circumference and sum of subscapular and triceps skinfolds were also available for investigation.

### Covariates

Important covariates were identified and harmonized for subsequent analyses. These included maternal height (in cm), prepregnancy body mass index (BMI; in kg/m^2^), maternal age at delivery (in year), maternal education status (study-specific definition of low/medium/high), self-reported maternal birth place/maternal ethnicity (European-born/white or non-European-born/non-white), maternal cigarette smoking (never/ever/current), maternal alcohol intake during pregnancy (yes/no), maternal parity (primiparous/multiparous), and infant sex (male/female). These data were originally collected by questionnaires (interviewer- or self-administered) or abstracted from birth records. Data are expressed in different units and categories in different studies, thus we harmonized the covariates across studies through standardizing units and categories for downstream analysis. For example, educational attainment was recategorized based on study-specific definitions of low, medium, and high. In addition, some countries did not allow specific question on “ethnicity” and thus can only be proxied by questions on place of birth. We grouped participants who specifically reported as being of European ancestry and those reported as being born in Europe into 1 group.

### Statistical analysis

Participants’ characteristics were summarized for the ALPHABET consortium and according to its constituent studies. These were limited to participants with availability of the exposure (maternal diet) and main outcomes (birth weight or gestational age) information. We further excluded participants with implausible energy intakes (<500 or >3,500 kcal/d) to avoid extreme misreporting [47,48].

A 2-stage individual participant data meta-analysis was used to assess the associations between maternal diet quality and inflammatory potential and birth outcomes. Cohort-specific effect estimates were first obtained by using linear and logistic regressions for continuous and binary outcomes, respectively. The effect estimates were then pooled using random-effects meta-analysis following methods described by DerSimonian and Laird [49], which considers both within- and between-study variability. Statistical heterogeneity among included studies was assessed using the Cochran Q test and *I*^2^-statistic [50,51].

Based on literature, the following set of a priori selected covariates were adjusted for: maternal education, ethnicity, prepregnancy BMI, maternal height, parity, energy intake for DASH, cigarette smoking and alcohol consumption during pregnancy, and infant sex. All the selected covariates were statistically significantly different across quartiles of DASH and E-DII, except for the E-DII–prepregnancy BMI relationship. Missing covariates information was imputed using the cohort-specific means for continuous variables and the modal categories for categorical variables. Complete case analysis yielded largely similar results and did not affect study conclusions (S5 and S6 Tables).

We also conducted several sensitivity analyses. First, we limited our analysis to European-born/white participants. Second, we excluded participants with gestational diabetes, gestational hypertension, and preeclampsia. Third, for gestational age outcomes, we restricted the analysis sample to spontaneous labors to exclude influence of elective procedures. Fourth, for primary birth size measures, we restricted the analysis sample to term (born between 37 to <42 completed weeks) and full-term (39 to <41 completed weeks) infants. For continuous birth size measures (birth weight, birth length, and head circumference), we also further adjusted for gestational age to assess potential mediation. Additionally, we also assessed whether imputation methods affected our results by conducting a sensitivity analysis using multiple imputation of covariates during the prepregnancy and whole pregnancy periods. Chained equation [52] was used to impute the missing covariates 10 times (separately in each cohort), resulting in 10 imputed analytic datasets, of which resulting regression coefficients were subsequently pooled. To explore potential mechanism, we mutually adjusted for DASH and E-DII scores in the same model. To investigate potential threshold influence, we also modeled the dietary scores in quartiles for statistically significant associations.

We investigated whether infant sex is a potential modifier for the associations of maternal dietary quality and inflammatory potential with birth outcomes by including the multiplicative interaction term into the model one at a time. This was done using participant-level data at each cohort, and the within-cohort interaction estimates were subsequently pooled. When *P*-interaction was ≤0.10, downstream subgroup stratification analyses were also conducted to aid visualization.

All analyses were performed using the statistical software Stata version 13.1 (StataCorp, College Station, Texas, United States of America), and statistical significance was defined as 2-sided *P* values < 0.05.

## Results

**Fig 1:**
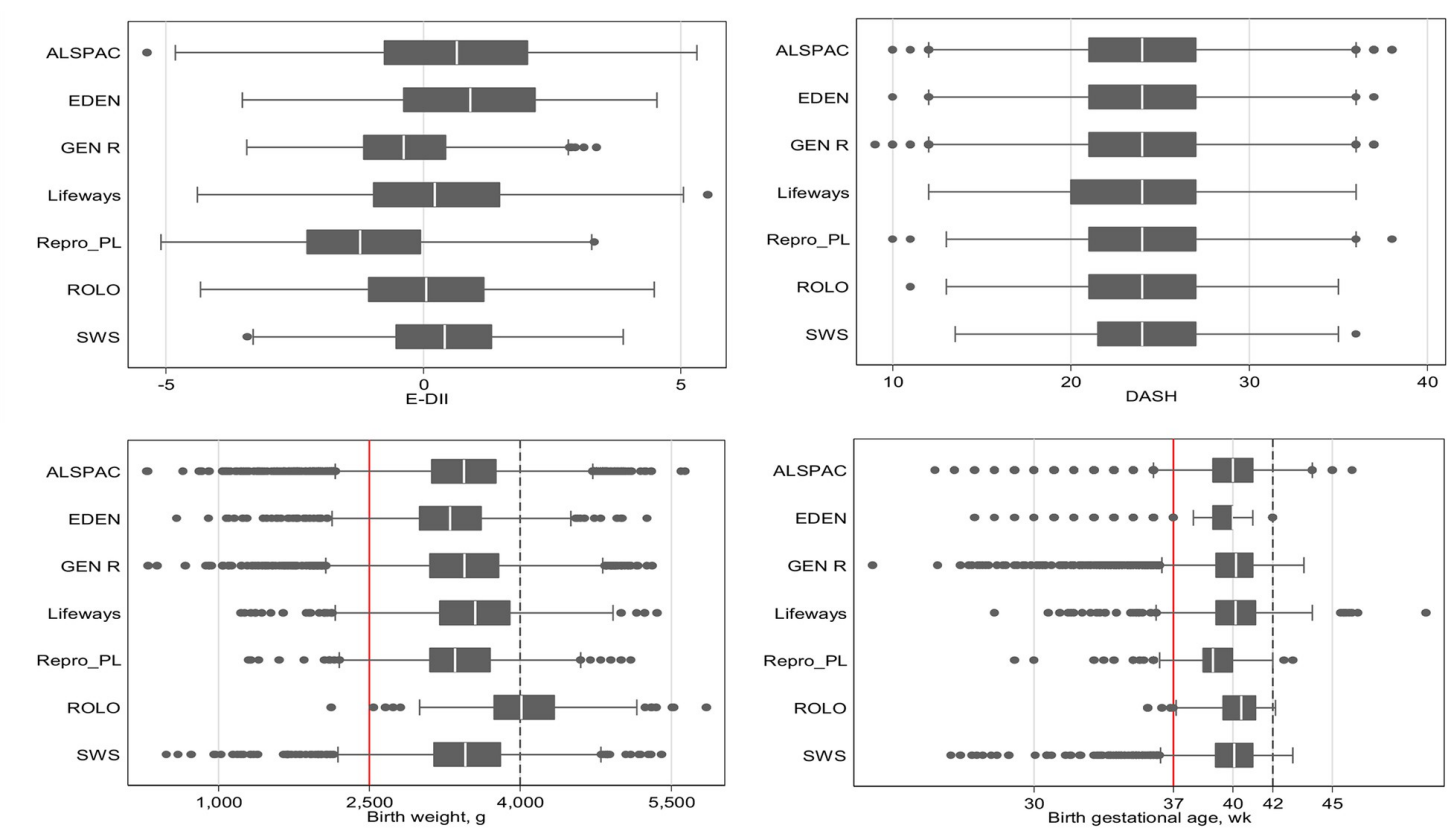
Boxplots of maternal pregnancy E-DII and DASH scores and offspring birth weight and gestational age at birth according to constituent studies. The red vertical solid and dotted lines represent clinical cutoffs for adverse birth outcomes. DASH, Dietary Approaches to Stop Hypertension; E-DII, energy-adjusted Dietary Inflammatory Index.

The current analysis included up to 24,861 mother–child pairs from 7 European studies. Overall, the study mothers had a mean ± SD age of 29.5 ± 4.9 y at delivery and a mean BMI of 23.3 ± 4.2 kg/m^2^ (S1 Table). Mean ± SD of dietary scores were: prepregnancy E-DII = 0.1 ± 1.7; pregnancy E-DII = 0.2 ± 1.7; prepregnancy DASH = 24.1 ± 4.3; pregnancy DASH = 24.0 ± 4.2. The Pearson’s correlations coefficients between E-DII and DASH were −0.60 for prepregnancy and −0.49 during pregnancy (both *P* <0.001). Most of the mothers reported European-born/white ethnicity. There were notable differences in participants’ characteristics across included studies, despite all being set in Europe. For example, about one-third of Generation R’s participants self-reported to be of non-European-born/non-white ethnicity, in contrast to <5% in other cohorts. The distributions of main exposure (maternal E-DII and DASH scores during pregnancy) and outcomes (birth weight and gestational age) are shown in Fig 1.

**Fig 2:**
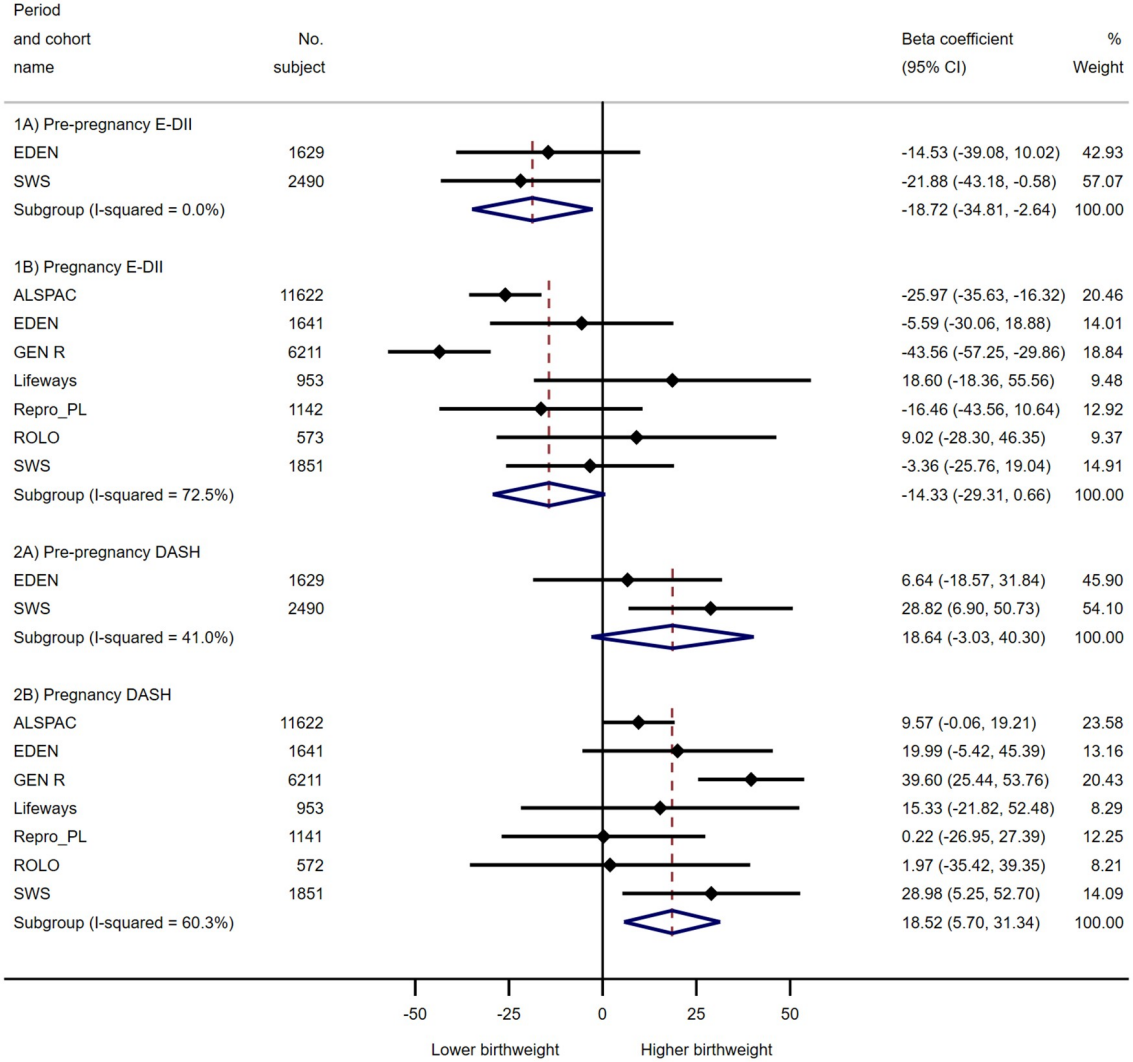
Forest plot showing adjusted associations of maternal E-DII and DASH scores (per 1-SD increase) during prepregnancy and pregnancy periods with birth weight. Black dots indicate study-specific point effect estimates with corresponding 95% CIs indicated by horizontal lines, and diamonds indicate the pooled estimates with their corresponding 95% CIs. When studies were omitted one at a time for pregnancy DASH meta-analysis, the overall pooled estimates remained largely the same and remained statistically significant: range of beta coefficients = 11.7 to 21.6 g, all CIs did not include 0; for E-DII, beta coefficients (95% CI) range from −8.8 (−23.0, 5.5) g when excluding Gen R to −18.3 (−32.8, −3.8) g when excluding Lifeways. Effect estimates were adjusted for maternal education, ethnicity, prepregnancy BMI, maternal height, parity, energy intake (for DASH), cigarette smoking and alcohol consumption during pregnancy, and child sex. BMI, body mass index; DASH, Dietary Approaches to Stop Hypertension; E-DII, energy-adjusted Dietary Inflammatory Index.

**Fig 3:**
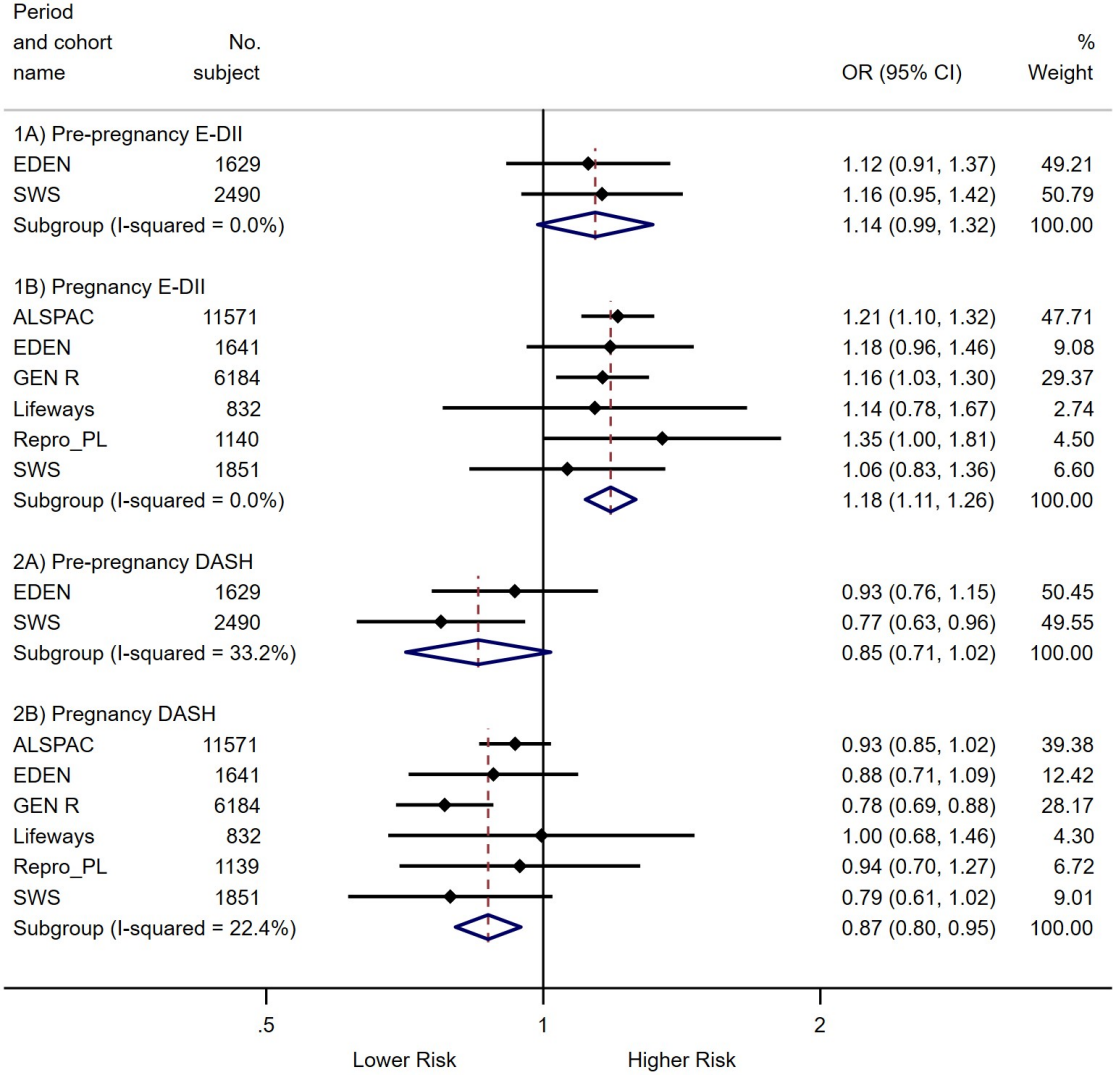
Forest plot showing adjusted associations of maternal E-DII and DASH scores (per 1-SD increase) during prepregnancy and pregnancy periods with risk of SGA. Black dots indicate study-specific point effect estimates with corresponding 95% CIs indicated by horizontal lines, and diamonds indicate the pooled estimates with their corresponding 95% CIs. As the ROLO study has estimates with very wide confidence intervals that impaired data visualization, it was excluded from this figure. By design, the ROLO study recruited mothers who have previously delivered a macrosomic infant, thus infants born low birth weight or SGA in this study were rare (<1%), causing unstable estimates. However, it should be noted that excluding this study did not affect the pooled estimates and overall conclusion [pooled OR (95% CI) = 1.18 (1.11, 1.26) for E-DII and 0.87 (0.80, 0.95) for DASH when ROLO was excluded cf. pooled OR (95% CI) = 1.18 (1.11, 1.26) for E-DII and 0.87 (0.82, 0.94) for DASH when ROLO was included]. When other studies were omitted one at a time for pregnancy E-DII and DASH meta-analysis, the overall pooled estimates were not affected and remained statistically significant: for E-DII: (range of ORs = 1.16–1.19, all CIs did not include 1); for DASH: (range of ORs: 0.82–0.92, all CIs did not include 1). Effect estimates were adjusted for maternal education, ethnicity, prepregnancy BMI, maternal height, parity, energy intake (for DASH), cigarette smoking and alcohol consumption during pregnancy, and child sex. BMI, body mass index; DASH, Dietary Approaches to Stop Hypertension; E-DII, energy-adjusted Dietary Inflammatory Index; OR, odds ratio; ROLO, Randomised cOntrol trial of LOw glycaemic index diet during pregnancy study; SGA, small-for-gestational-age.

The associations of maternal prepregnancy and pregnancy E-DII and DASH scores with offspring birth outcomes are documented in Table 1 (continuous measures) and Table 2 (binary outcomes). Overall, a more pro-inflammatory and a lower-quality maternal diet during pregnancy were associated with lower birth size measures and higher risk of giving birth to LBW or SGA infants. Higher maternal prepregnancy E-DII score (more pro-inflammatory diet) was associated with lower birth weight [β (95% CI) = −18.7 (−34.8, −2.6) g per 1-SD increase in maternal E-DII score; *P* value = 0.023] and shorter birth length [−0.07 (−0.14, −0.01) cm; *P* value = 0.031], whereas higher pregnancy E-DII score was associated with a shorter birth length [−0.06 (−0.10, −0.01) cm; *P* value = 0.031] and a higher risk of LBW [odds ratio (OR) (95% CI) = 1.14 (1.04, 1.26); *P* value = 0.008] and SGA [1.18 (1.11, 1.26); *P* value < 0.001]. No consistent association was observed between prepregnancy DASH and primary continuous and binary outcomes (both birth weight and gestational age). However, higher maternal pregnancy DASH score (higher dietary quality) was associated with higher birth weight [β (95% CI) = 18.5 (5.7, 31.3) g per 1-SD increase in DASH score; *P* value = 0.005], longer birth length [0.05 (0.01, 0.10) cm; *P* value = 0.010], higher head circumference [0.03 (0.01, 0.06) cm; *P* value = 0.004], and lower risk of delivering LBW [OR (95% CI) = 0.89 (0.82, 0.95); *P* value = 0.001] and SGA [0.87 (0.82, 0.94); *P* value < 0.001] infants. These were consistently observed for early-pregnancy and late-pregnancy DASH scores. Maternal E-DII and DASH scores were not associated with indicators of excessive fetal growth (macrosomia and LGA). The main results for birth weight and risk of SGA were summarized in the form of forest plots in Figs 2 and 3.

**Table 1:** Association between maternal E-DII and DASH scores (per 1-SD increase) and continuous offspring outcomes in the ALPHABET consortium.

	Primary outcomes				Secondary outcomes							
	Birthweight, g		Gestational age, wk		Birth length, cm		Head circumference, cm		Abdominal circumference, cm		Sum of skinfold thickness, mm	
	β (95% CI)	*I*^*2*^ *(%)*	β (95% CI)	*I*^*2*^ *(%)*	β (95% CI)	*I*^*2*^ *(%)*	β (95% CI)	*I*^*2*^ *(%)*	β (95% CI)	*I*^*2*^ *(%)*	β (95% CI)	*I*^*2*^ *(%)*
**E-DII**												
*Pre*	−18.7 (−34.8, −2.6)	0	−0.02 (−0.08, 0.04)	0	−0.07 (−0.14, −0.01)	0	−0.04 (−0.10, 0.03)	43	−0.01 (−0.09, 0.08)	-	−0.03 (−0.09, 0.03)	0
*P value*	0.023	0.66	0.52	0.46	0.031	0.64	0.27	0.18	0.82	-	0.30	0.39
Np/Nc	4,119/2		4,137/2		3,964/2		3,993/2		2,406/1		3,923/2	
*Preg*	−14.3 (−29.3, 0.7)	73	−0.02 (−0.07, 0.03)	72	−0.06 (−0.10, −0.01)	34	−0.03 (−0.07, 0.01)	54	0.05 (−0.10, 0.20)	31	−0.02 (−0.10, 0.06)	25
*P value*	0.06	0.001	0.47	0.001	0.026	0.17	0.09	0.045	0.51	0.23	0.60	0.26
Np/Nc	23,993/7		24,101/7		18,953/7		18,333/7		2,031/2		3,531/3	
*Early*	−12.1 (−36.0, 11.9)	77	−0.03 (−0.10, 0.04)	70	−0.05 (−0.13, 0.03)	46	−0.03 (−0.09, 0.04)	66	0.04 (−0.15, 0.24)	48	−0.12 (−0.34, 0.10)	44
*P value*	0.32	0.002	0.43	0.010	0.19	0.12	0.41	0.019	0.66	0.16	0.30	0.18
Np/Nc	10,863/5		10,825/5		8,381/5		7,640/5		2,129/2		2,082/2	
*Late*	−15.2 (−30.7, 0.3)	58	0.004 (−0.06, 0.07)	75	−0.05 (−0.09, −0.01)	0	−0.03 (−0.06, −0.01)	0	−0.02 (−0.10, 0.07)	-	0.01 (−0.04, 0.07)	0
*P value*	0.06	0.09	0.90	0.019	0.015	0.56	0.016	0.51	0.70	-	0.66	0.77
Np/Nc	15,621/3		15,784/3		12,955/3		13,104/3		2,309/1		3,842/2	
**DASH**												
*Pre*	18.6 (−3.0, 40.3)	41	0.001 (−0.06, 0.06)	0	0.07 (0.004, 0.14)	0	0.01 (−0.04, 0.06)	0	0.10 (0.01, 0.18)	-	0.06 (−0.004, 0.13)	25
*P value*	0.09	0.19	0.97	0.36	0.039	0.62	0.70	0.60	0.033	-	0.06	0.25
Np/Nc	4,119/2		4,137/2		3,964/2		3,993/2		2,406/1		3,923/2	
*Preg*	18.5 (5.7, 31.3)	60	0.02 (-0.01, 0.05)	33	0.05 (0.01, 0.10)	15	0.03 (0.01, 0.06)	0	0.01 (-0.22, 0.23)	56	0.06 (-0.01, 0.13)	11
*P value*	0.005	0.020	0.18	0.18	0.010	0.32	0.004	0.59	0.97	0.13	0.08	0.33
Np/Nc	23,991/7		24,100/7		18,952/7		18,333/7		2,030/2		3,530/3	
*Early*	20.9 (3.3, 38.5)	55	0.03 (−0.02, 0.07)	40	0.09 (0.03, 0.14)	0	0.04 (0.001, 0.07)	0	0.002 (−0.21, 0.21)	52	0.12 (0.03, 0.20)	0
*P value*	0.020	0.06	0.31	0.16	0.001	0.68	0.047	0.46	0.99	0.15	0.007	0.92
Np/Nc	10,861/5		10,824/5		8,380/5		7,640/5		2,128/2		2,081/2	
*Late*	16.9 (4.0, 29.9)	40	0.02 (−0.01, 0.05)	2	0.05 (−0.02, 0.12)	55	0.03 (0.01, 0.06)	0	0.11 (0.02, 0.20)	-	0.04 (−0.02, 0.10)	0
*P value*	0.010	0.19	0.16	0.36	0.13	0.11	0.012	0.47	0.019	-	0.19	0.44
Np/Nc	15,620/3		15,783/3		12,954/3		13,103/3		2,308/1		3,841/2	

Values are adjusted pooled effect estimates [β (95% CI)] expressed for a 1-SD increment in dietary scores, heterogeneity measure (*I*^*2*^), and number of participants and studies included (Np/Nc) across different outcomes and conception periods, as labeled. Effect estimates were adjusted for maternal education, ethnicity, prepregnancy BMI, maternal height, parity, energy intake (for DASH), cigarette smoking and alcohol consumption during pregnancy, and child sex.

DASH, Dietary Approaches to Stop Hypertension; Early, early pregnancy; E-DII, energy-adjusted Dietary Inflammatory Index; *I*^2^, *I*-squared; Late, late pregnancy;; Nc, number of cohorts included; Np, number of participants included; Pre, prepregnancy; Preg, pregnancy.

**Table 2:** Association between maternal E-DII and DASH scores (per 1-SD increase) and binary offspring outcomes in the ALPHABET consortium.

	Low birth weight		SGA		Macrosomia		LGA		Preterm birth		Postterm birth	
	OR (95% CI)	*I*^*2*^ *(%)*	OR (95% CI)	*I*^*2*^ *(%)*	OR (95% CI)	*I*^*2*^ *(%)*	OR (95% CI)	*I*^*2*^ *(%)*	OR (95% CI)	*I*^*2*^ *(%)*	OR (95% CI)	*I*^*2*^ *(%)*
**E-DII**												
Pre	1.16 (0.99, 1.36)	0	1.14 (0.99, 1.32)	0	0.99 (0.89, 1.10)	0	0.97 (0.89, 1.05)	0	1.04 (0.90, 1.19)	0	1.10 (0.92, 1.31)	0
*P value*	0.06	0.73	0.08	0.81	0.79	0.81	0.41	0.83	0.60	0.44	0.31	0.38
Np/Nc	4,119/2		4,119/2		4,065/2		4,119/2		4,137/2		4,137/2	
Preg	1.14 (1.04, 1.26)	27	1.18 (1.11, 1.26)	0	0.95 (0.89, 1.02)	49	0.98 (0.92, 1.05)	63	1.02 (0.92, 1.13)	40	0.99 (0.92, 1.06)	15
*P value*	0.008	0.23	<0.001	0.93	0.14	0.07	0.58	0.012	0.75	0.12	0.75	0.31
Np/Nc	23,571/7		23,370/7		23,938/7		23,791/7		23,953/7		23,998/7	
Early	1.19 (1.08, 1.32)	0	1.17 (1.07, 1.29)	0	0.99 (0.90, 1.09)	52	1.02 (0.91, 1.13)	72	1.10 (1.003, 1.21)	0	1.01 (0.92, 1.10)	0
*P value*	0.001	0.97	0.001	0.91	0.85	0.08	0.75	0.007	0.042	0.74	0.89	0.81
Np/Nc	10,441/5		10,291/5		10,863/5		10,712/5		10,677/5		10,722/5	
Late	1.04 (0.84, 1.30)	72	1.18 (1.09, 1.28)	0	0.93 (0.83, 1.04)	59	0.95 (0.90, 1.01)	22	0.97 (0.82, 1.16)	66	1.07 (0.87,1.31)	68
*P value*	0.70	0.029	<0.001	0.50	0.19	0.09	0.07	0.28	0.74	0.05	0.51	0.044
Np/Nc	15,621/3		15,570/3		15,566/3		15,570/3		15,784/3		15,784/3	
**DASH**												
Pre	0.88 (0.61, 1.28)	81	0.85 (0.71, 1.02)	33	1.05 (0.94, 1.17)	0	1.05 (0.92, 1.20)	51	0.95 (0.82, 1.10)	0	0.90 (0.67, 1.20)	50
*P value*	0.51	0.023	0.08	0.22	0.43	0.61	0.44	0.16	0.49	0.51	0.47	0.16
Np/Nc	4,119/2		4,119/2		4,065/2		4,119/2		4,137/2		4,137/2	
Preg	0.89 (0.82, 0.95)	0	0.87 (0.82, 0.94)	7	1.03 (0.97, 1.10)	35	1.05 (0.99, 1.12)	48	0.96 (0.89, 1.04)	13	0.98 (0.89, 1.08)	38
*P value*	0.001	0.49	<0.001	0.38	0.30	0.16	0.08	0.07	0.33	0.33	0.62	0.14
Np/Nc	23,570/7		23,369/7		23,936/7		23,790/7		23,952/7		23,997/7	
Early	0.83 (0.75, 0.92)	0	0.82 (0.74, 0.90)	0	1.04 (0.97, 1.11)	14	1.05 (0.97, 1.13)	41	0.98 (0.85, 1.13)	36	0.96 (0.85, 1.07)	14
*P value*	0.001	0.46	<0.001	0.66	0.28	0.32	0.22	0.15	0.79	0.18	0.44	0.33
Np/Nc	10,440/5		10,290/5		10,861/5		10,711/5		10,676/5		10,721/5	
Late	0.92 (0.84, 1.002)	0	0.87 (0.77, 0.99)	43	1.07 (0.95, 1.19)	57	1.09 (0.99, 1.20)	66	0.95 (0.88, 1.03)	0	0.93 (0.76, 1.13)	64
*P value*	0.06	0.87	0.037	0.18	0.27	0.10	0.10	0.06	0.20	0.54	0.46	0.06
Np/Nc	15,620/3		15,569/3		15,565/3		15,569/3		15,783/3		15,783/3	

Values are adjusted pooled effect estimates [OR (95% CI)] expressed for a 1-SD increment in dietary scores, heterogeneity measure (*I*^2^), and number of participants and studies included (Np/Nc) across different outcomes and conception periods, as labeled. Effect estimates were adjusted for maternal education, ethnicity, prepregnancy BMI, maternal height, parity, energy intake (for DASH), cigarette smoking and alcohol consumption during pregnancy, and child sex.

DASH, Dietary Approaches to Stop Hypertension; Early, early pregnancy; E-DII, energy-adjusted Dietary Inflammatory Index; *I*^2^, *I*-squared; Late, late pregnancy; LGA, large-for-gestational-age; Nc, number of cohorts included; Np, number of participants included; OR, odds ratio; Pre, prepregnancy; Preg, pregnancy; SGA, small-for-gestational-age.

In contrast, no consistent associations were observed between maternal E-DII, DASH and gestational age at birth (Table 1), and risk of delivering preterm and postterm infants (Table 2), except that higher early-pregnancy E-DII score was associated with a higher risk of preterm birth [OR (95% CI) = 1.10 (1.003, 1.21); *P* value = 0.042]. In a small subset of studies (EDEN, ROLO, SWS), higher maternal prepregnancy and late-pregnancy DASH scores were associated with higher offspring abdominal circumference [only 1 study (SWS) included], while higher early-pregnancy DASH score was associated with thicker offspring skinfolds, but these associations should be interpreted with caution due to smaller numbers.

In sensitivity analyses restricted to European-born/white participants (S7 and S8 Tables), participants without pregnancy complications (S9 and S10 Tables), spontaneous labors (S11 Table), and term and full-term infants (S12 and S13 Tables), the aforementioned results appeared robust and main conclusions remained unchanged. When gestational age was adjusted, the estimates for continuous birth size measures changed little (S14 Table). Results were also essentially the same when missing covariates were multiply imputed (S15 Table). When E-DII and DASH were mutually adjusted, statistical significances and point estimates for birth size measures attenuated appreciably for both dietary scores (S16 and S17 Tables). Of the significant associations reported in Tables 1 and 2, only 3 relationships showed evidence of departure from linearity: early-pregnancy DASH versus LBW, prepregnancy DASH versus birth length, and prepregnancy DASH versus abdominal circumference (*P* for quadratic term = 0.007, 0.031, and 0.001, respectively). Upon closer inspection, the influence of higher prepregnancy DASH versus longer birth length seemed to level off at the highest quartile, whereas higher prepregnancy DASH versus higher abdominal circumference and higher early-pregnancy DASH versus lower risk of LBW relationship appeared to level off after the second quartile, as compared with those in lowest quartile (see S18 Table for estimates).

**Fig 4:**
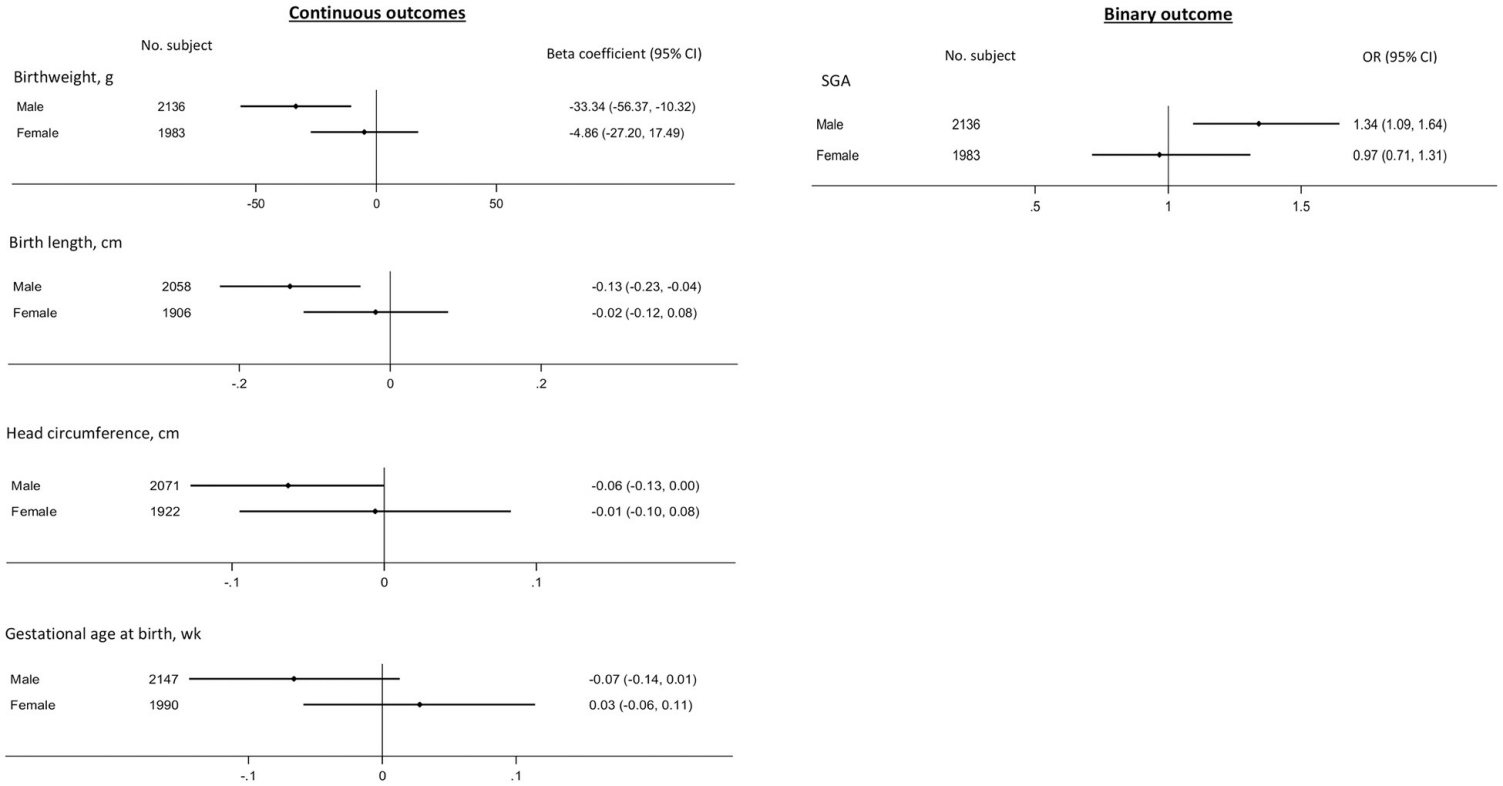
Forest plot showing sex-interaction between maternal prepregnancy E-DII score (per 1-SD increase) and offspring birth outcomes (only adjusted subgroup pooled estimates shown; all pooled *P* values of interaction term ≤0.10). The pooled estimates shown were based on analyses conducted separately in males and females to aid visualization; for this downstream subgroup analyses to be conducted, all *P* values were ≤0.10 based on meta-analysis of within-cohort interaction estimates.

We noted sex-interaction mainly between E-DII (especially prepregnancy) and birth size measures (see S19 Table for a compilation of *P*-interactions). Higher maternal prepregnancy E-DII was associated with lower birth weight and head circumference, shorter birth length, and a higher risk of SGA in male but not female infants (Fig 4). For pregnancy period, higher maternal E-DII also seemed to affect male birth size (lower birth weight) more than female (S20 Table). No apparent sex-interaction was observed for maternal DASH and birth outcomes association, except for prepregnancy DASH-SGA relationship, which also appeared stronger in male infants [OR (95% CI): 0.83 (0.70, 0.99) in male and 0.91 (0.73, 1.12) in female; *P*-interaction = 0.094].

## Discussion

In this large collaborative effort using harmonized individual participants data, we observed that, independent of prepregnancy BMI and other socioeconomic factors, a lower quality and more pro-inflammatory maternal diet was associated with smaller birth sizes (lower birth weight and shorter birth length) and a higher risk of delivering SGA infants. Furthermore, we observed interesting sex differences in the associations between maternal dietary inflammatory potential and birth outcomes. To our knowledge, this is hitherto the largest multicenter study confirming that maternal whole diet is associated with birth size (a proxy of fetal growth).

Our results appeared robust across several sensitivity analyses, including in a healthier sample of mothers without pregnancy complications or among full-term infants. In addition, further adjustment for gestational age had minimal influence on effect estimates, implying that the observed relationships between maternal dietary scores and birth size were not mediated through length of gestational duration. However, mutual adjustment of E-DII and DASH appreciably, but not completely, attenuated the associations, suggesting that the associations of general healthy eating with higher birth sizes might not have been mediated completely through lowering maternal inflammation, and other biological mechanisms such as epigenetic programming might be in play.

### Results in the context of other studies

There have been recent comprehensive systematic reviews (without [20] or with [21] meta-analysis) on maternal dietary patterns and birth outcomes. These reviews were, however, limited by the use of different techniques to define “healthy dietary pattern” in the original studies (data-driven, i.e., using dimension-reducing statistical techniques, such as factor analysis, or based on preexisting dietary indices such as Mediterranean diet and Healthy Eating Index). The substantial clinical heterogeneity has impeded firm conclusions concerning the influence of maternal dietary quality on birth outcomes. In the review with aggregate data meta-analysis, a “weak trend” was observed between greater adherence to “healthy dietary pattern” during pregnancy and lower risk of SGA/LBW/fetal growth restriction based on 10 studies [OR (95% CI) = 0.86 (0.73, 1.01) comparing extreme tertiles or “per 2.18 SD” cf. 0.74 (0.65, 0.87) for the same increment in ALPHABET]. In their subgroup analysis for studies examining SGA specifically (*n* = 5), the pooled estimate was statistically significant [OR (95% CI) = 0.91 (0.84, 0.97)]. Our results showed more consistent associations between maternal dietary quality and birth size, probably due to more consistent definition of healthy dietary pattern, harmonized covariates derivation, and analysis strategy. In contrast, both previous reviews noted limited but consistent evidence that maternal healthy eating during pregnancy was associated with a lower risk of preterm birth, as opposed to our observed null association between higher DASH score and preterm birth risk. It may be that the prevalence of preterm birth is too low in our study (<5%) or factors other than maternal dietary quality have a greater influence on the risk of preterm birth in developed countries with good access to healthcare. Interestingly, in subgroup analysis of the previous review, the association between maternal healthy eating during pregnancy and lower risk of preterm birth also appeared stronger in non-European versus European studies [21].

Limited studies have investigated maternal dietary inflammatory index and offspring birth outcomes, with conflicting results reported [53–55]. In concordance with our results, a US study observed that higher maternal DII score during pregnancy was associated with lower birth weight and higher risk of SGA among obese women [53], but no associations were apparent for gestational-age-related outcomes. In contrast, another US study reported higher offspring birth weight and a higher risk of LGA (but not SGA) with a higher maternal DII score [54]. Yet, another US study with a large proportion of African-American observed no association between maternal E-DII score and birth weight, SGA, and LGA [55]. These discrepancies in results could be potentially attributed to different ethnic composition, use of either DII or E-DII, and different population baseline dietary inflammatory potential. To the best of our knowledge, our study is the first to show an association between prepregnancy dietary inflammatory potential and smaller birth sizes, highlighting the importance of extending dietary advice issuance to the prepregnancy period.

We observed that child sex modifies the relationship between prepregnancy maternal dietary inflammatory potential with birth size. The inflammatory environment induced by pregnancy likely differs according to stages of pregnancy. The periconceptional phase, characterized by implantation and placentation, can be considered a pro-inflammatory phase [56]. It also has been shown that the male fetus induces a much more pro-inflammatory immune milieu than the female fetus at multiple time points during pregnancy [57]. Thus, the influence of a more pro-inflammatory periconceptional diet might be compounded by the immune response induced by the male fetus, resulting in impaired fetal growth for mothers who habitually consumed a more pro-inflammatory diet. In a recent systematic review and meta-analysis on sexual dimorphism in maternal pregnancy complications, the occurrence of most pregnancy complications, especially gestational diabetes and term preeclampsia, was found to be higher among women bearing male offspring [58]. This could be due to a higher cardiovascular and metabolic load for the mother carrying male fetus [58]. In addition, high maternal BMI and gestational diabetes, both related to adverse maternal metabolic health, have also been shown to be associated with higher risk of macrosomia only among male infants [59]. Taken together and pending confirmation in future mechanistic studies, observations from our and other studies that a pro-inflammatory diet and suboptimal maternal metabolic health may negatively affect male fetus more prominently could highlight the need to intensify efforts to reduce dietary inflammatory potential among women bearing a male fetus. However, since we also observed significant associations in the overall population, a healthy, anti-inflammatory pregnancy diet is likely beneficial for the female fetus too and thus should not be overlooked.

### Strengths and limitations of study

Our study was strengthened by the large sample size and substantial efforts spent in harmonizing and curating data across multiple studies. Because we included only prospective studies, the temporal sequence between maternal diet and birth outcomes can be established. Furthermore, we were able to adjust for a comprehensive range of covariates not considered in previous studies [20].

Despite our study’s obvious strengths, its findings should be interpreted with some caution. Although our consortium included studies in a range of geographical regions within Europe (British Isles, Western and Eastern Continental Europe) having some differences in dietary intakes and sociodemographic characteristics, our study samples can mainly be generalized to white women in developed countries. Despite substantial efforts spent to reduce clinical heterogeneity by harmonizing data across included studies, especially for a harmonized definition of general healthy eating and inflammatory potential, it should be kept in mind that some pooled analyses were associated with high statistical heterogeneity (in Tables 1 and 2, percentage analyses with *I*^*2*^*>*50% = 26%; mostly associated with nonstatistically significant pooled estimates). This could be due to residual clinical and methodological heterogeneity, or that the true effects may vary from study to study—an assumption of our chosen random-effects model. To further reduce heterogeneity, a prospectively planned IPD with harmonized study design should be conducted. Dietary data were self-reported (though with mostly validated questionnaires) before the outcomes, which might have increased nondifferential measurement errors that may bias results toward the null. Diet measures are based on FFQs with different degree of detail (number of food items, response categories, etc.) (S3 Table), which could result in discrepancies in estimated intakes and diet scores. However, generation of E-DII and the DASH scores involved 20 to 28 (out of 44 possible) dietary parameters, and 48.1% to 79.1% of the total FFQ food items, respectively. Concerning the E-DII, previous studies have found adequate predictive ability with as few as 18 parameters [60], while most food component of the DASH score comprised at least 5 food items [40]. While a prospectively planned consortium study with harmonized dietary assessment method would be ideal to further reduce methodological variation, we believe that the instruments used by the respective studies in ALPHABET are sufficient to capture the variation in dietary quality and inflammation. Furthermore, prevalence rates for the main adverse birth outcomes were low (e.g., <5% for LBW and preterm birth). In populations with an overall poorer-quality diet and higher prevalence of fetal growth restriction, our estimates could be an underestimation of actual effect. We did not observe associations between maternal dietary quality and inflammatory potential and postterm delivery. Similar to preterm birth, the proportion of infants delivered postterm was quite low (6.5%; see S4 Table), and our study might have been similarly underpowered to detect any associations. Furthermore, postterm delivery could be heavily influenced by external factors such as clinician behavior and institutional culture. Although we similarly observed no associations among spontaneous labors only (75% of postterm delivery was spontaneous), our results should be confirmed in future studies with better information on practice in different health systems. We explored stratified analysis in Generation R with a significant proportion of non-European-born/non-white participants (S21 Table). While the higher dietary inflammatory potential versus lower birth sizes and higher preterm birth risk relationships seemed to be more pronounced in non-European-born/non-white participants in Generation R, overall the effect estimates associated with DASH score seemed to be closer to the null, as compared with European-born/white participants. However, it should be noted that the non-white group is not a homogenous group and that the migration history/demography differs in each country. Thus, the relationship between maternal diet and birth outcomes should be investigated further in other homogenous populations or studies with more specific ethnicity information. Control of some potentially confounding variables could be less precise in this study due to the need to harmonize the variables across cohorts. Finally, as with any observational study, causality cannot be established without corroborating evidence, and the influence of residual confounding cannot be completely ruled out.

### Conclusions and public health implications

Our findings may have important clinical and public health implications. For example, a higher maternal dietary quality indicated by higher maternal pregnancy DASH score, scaled to a very plausible 2-SD increase (8.4 points increment; ALPHABET range: 9 to 38), was associated with a 37 g higher birth weight and a 24% lower risk in delivering SGA infants. Considering the high prevalence of fetal growth restriction, especially in developing countries, and its potential negative impacts on lifelong health, improving overall maternal dietary quality is of utmost importance. Our findings represent a significant contribution to the knowledge base regarding the importance of maternal diet on offspring health outcomes. Better understanding of these relationships may inform revision of existing dietary guidelines or development of new guidelines for optimal nutrition in pregnancy. Policies to ensure availability of affordable healthy foods and programmatic efforts to inform and support women of reproductive age, such as raising awareness of the importance of maternal diet and prenatal and antenatal counseling, would help women achieve a healthier diet.

In conclusion, maternal diet that is of low quality and high inflammatory potential is associated with lower offspring birth size and higher risk of offspring being born SGA in this multicenter meta-analysis using harmonized individual participants data. Although confirmation from other sources including randomized controlled trials are needed to establish causality, our results strongly suggest that improving overall maternal dietary pattern based on predefined criteria related to overall quality and inflammatory potential is beneficial for optimal fetal growth.

## Supporting information

S1 Text Cohort-specific sources of funding. (DOCX)

S2 Text Project protocol for funding application. (DOCX)

S1 Table Characteristics of study participants according to included studies. (DOCX)

S2 Table Information on data request contacts, full cohort recruitment date, and local institutional ethical review boards for each cohort. (DOCX)

S3 Table Food Frequency Questionnaires and Diet scores in the ALPHABET consortium. (DOCX)

S4 Table Proportions of infants with adverse birth outcomes. (DOCX)

S5 Table Sensitivity analysis for continuous outcomes—complete case analysis. (DOCX)

S6 Table Sensitivity analysis for binary outcomes—complete case analysis. (DOCX)

S7 Table Sensitivity analysis for continuous outcomes—excluding all non-European-born/non-white participants. (DOCX)

S8 Table Sensitivity analysis for binary outcomes—excluding all non-European-born/non-white participants. (DOCX)

S9 Table Sensitivity analysis for continuous outcomes—excluding participants with pregnancy complications. (DOCX)

S10 Table Sensitivity analysis for binary outcomes—excluding participants with pregnancy complications. (DOCX)

S11 Table Sensitivity analysis for gestational age outcomes—restricting samples to all spontaneous labors. (DOCX)

S12 Table Sensitivity analysis for primary birth size measure—restricting samples born between 37 to <42 completed weeks of gestational age. (DOCX)

S13 Table Sensitivity analysis for primary birth size measure—restricting samples born between 39 to <41 completed weeks of gestational age. (DOCX)

S14 Table Sensitivity analysis for continuous birth size measure not intrinsically adjusted for gestational age—further adjusting for gestational age to assess potential mediation. (DOCX)

S15 Table Comparison of results with different imputation methods for covariates. (DOCX)

S16 Table Sensitivity analysis for continuous outcomes—mutually adjusting for dietary scores. (DOCX)

S17 Table Sensitivity analysis for binary outcomes—mutually adjusting for dietary scores. (DOCX)

S18 Table Quartile estimates for nonlinear relationships. (DOCX)

S19 Table Pooled *P* values for sex-interaction between maternal dietary quality and inflammatory potential and offspring birth outcomes. (DOCX)

S20 Table Sex-interaction between maternal pregnancy E-DII score and offspring birth outcomes (all *P*-interactions ≤0.10). (DOCX)

S21 Table Association between maternal pregnancy E-DII and DASH scores (per 1-SD increase) and offspring (A) continuous outcomes and (B) binary outcomes in the Generation R study. (DOCX)

S1 PRISMA Checklist Checklist of items to include when reporting a systematic review and meta-analysis of individual participant data (IPD). (DOCX)

## Abbreviations

ALSPAC: Avon Longitudinal Study of Parents and Children
BMI: body mass index
DASH: Dietary Approaches to Stop Hypertension
DOHaD: Developmental Origins of Health and Diseases
E-DII: energy-adjusted Dietary Inflammatory Index
FFQ: food frequency questionnaire
IPD: individual participant data
LBW: low birth weight
LGA: large-for-gestational-age
PRISMA-IPD: Preferred Reporting Items for Systematic Reviews and Meta-Analyses of Individual Participants Data
ROLO: Randomised cOntrol trial of LOw glycaemic index diet during pregnancy study
SGA: small-for-gestational-age
SWS: Southampton Women’s Survey
