# Supplementary material for: Associations of maternal dietary inflammatory potential and quality with offspring birth outcomes: An individual participant data pooled analysis of 7 European cohorts in the ALPHABET consortium
Source: PLoS Med. 2021 Jan 21;18(1):e1003491. doi: 10.1371/journal.pmed.1003491 (PMC7819611; doi:10.1371/journal.pmed.1003491)
Supplement: S1 Text — (DOCX) [file pmed.1003491.s001.docx]

**S1 Text** Cohort-specific sources of funding

ALSPAC: The UK Medical Research Council and Wellcome (Grant ref: 102215/2/13/2) and the University of Bristol provide core support for ALSPAC. This publication is the work of the authors and MS will serve as guarantor for the contents of this paper. EDEN: The EDEN study was supported by Foundation for medical research (FRM), National Agency for Research (ANR), National Institute for Research in Public health (IRESP: TGIR cohorte santé 2008 program), French Ministry of Health (DGS), French Ministry of Research, INSERM Bone and Joint Diseases National Research (PRO-A), and Human Nutrition National Research Programs, Paris-Sud University, Nestlé, French National Institute for Population Health Surveillance (InVS), French National Institute for Health Education (INPES), the European Union FP7 programmes (FP7/2007–2013, HELIX, ESCAPE, ENRIECO, Medall projects), Diabetes National Research Program (through a collaboration with the French Association of Diabetic Patients (AFD)), French Agency for Environmental Health Safety (now ANSES), Mutuelle Générale de l’Education Nationale a complementary health insurance (MGEN), French national agency for food security, French-speaking association for the study of diabetes and metabolism (ALFEDIAM). Generation R: The Generation R Study is made possible by financial support from the Erasmus Medical Centre, Rotterdam, the Erasmus University Rotterdam and The Netherlands Organization for Health Research and Development. The project received funding from the European Union's Horizon 2020 research and innovation programme (LIFECYCLE project, grant agreement no 733206; 2016). The study sponsors had no role in the study design, data analysis, interpretation of data, or writing of this report. Lifeways: The Lifeways Cross-Generation Cohort Study is funded by the Irish Health Research Board (reference HRC/2007/13) and is overseen by an inter-disciplinary steering group. REPRO_PL: The REPRO_PL cohort was mainly supported by the Ministry of Science and Higher Education, Poland (PBZ-MEiN-/8/2/2006; contract no. K140/P01/2007/1.3.1.1); by the grant PNRF-218-AI-1/07 from Norway through the Norwegian Financial Mechanism within the Polish- Norwegian Research Fund and National Science Centre, Poland (DEC-2014/15/B/NZ7/00998). ROLO: ROLO study is supported by Health Research Board Health Research Centre for Diet and Health Research Ireland and The National Maternity Hospital Medical Fund, and The European Union’s Seventh Framework Programme (FP7/2007–2013). SWS: This work was supported by grants from the Medical Research Council, British Heart Foundation, Arthritis Research UK, Food Standards Agency, and the European Union’s Seventh Framework (FP7/2007–2013), projects EarlyNutrition and ODIN under grant agreement numbers 289346 and 613977. The funders had no role in study design, data collection and analysis, decision to publish, or preparation of the manuscript.
