# Supplementary material for: Associations of maternal dietary inflammatory potential and quality with offspring birth outcomes: An individual participant data pooled analysis of 7 European cohorts in the ALPHABET consortium
Source: PLoS Med. 2021 Jan 21;18(1):e1003491. doi: 10.1371/journal.pmed.1003491 (PMC7819611; doi:10.1371/journal.pmed.1003491)
Supplement: S2 Text — (DOCX) [file pmed.1003491.s002.docx]

**S2 Text** Project protocol for funding application

**WP 4: Adiposity and cardiometabolic health**

***Leader:*** *Dr. C Phillips, University College Dublin, Ireland;* ***Participants:*** *ALSPAC, EDEN, Generation R, Lifeways, PEARS, Repro_PL and ROLO.*

**Background:** Both low and high birth weight are associated with fetal and neonatal mortality and morbidity and increased risk of chronic diseases in later life. Although it is generally accepted that maternal diet before and during pregnancy could potentially play an important role in maternal and offspring health outcomes, the specific dietary requirements during pregnancy for optimal fetal growth and development are unknown. Little to no information exists regards maternal dietary quality or inflammatory potential in this context. The proposed research offers an exceptional opportunity to investigate associations between novel dietary indices (reflecting whole diet) during pregnancy and fetal, infant, childhood and adolescent anthropometric measures. Approximately one quarter of children worldwide are overweight or obese. This is particularly concerning as childhood obesity tracks to adulthood and is associated with increased risk of cardiometabolic disease and premature mortality. In recent years different sub-phenotypes of obesity have been described including metabolically healthy obesity (MHO), whereby a proportion of obese individuals despite excess body fat remain free of metabolic abnormalities and increased cardiometabolic risk. Our data from the HRB Centre for Health and Diet Research (Mitchelstown cohort) have shown that inflammatory status and dietary quality may determine metabolic health status among obese middle-aged adults. Data regards childhood MHO is scarce, but it has been suggested that the MHO phenotype starts in childhood and persists into adulthood. Investigating metabolic health and adiposity during childhood may open up new avenues in the context of preventative strategies for adverse metabolic health phenotypes in early life, which may have long term impacts. For example, identification of potential intervention windows and targets to improve cardiometabolic profiles in paediatric obesity with a view to achieving and maintaining better long term cardiometabolic health. Most maternal diet-offspring DNA methylation analyses to date have focussed on intake of selected micronutrients. The influence of maternal whole diet (assessed by DASH or DII) or dietary intervention during pregnancy (such as in the PEARs study) on the offspring epigenome and inter-relationships with adiposity/cardiometabolic health remains unknown and warrants further investigation. **Aims:** Building on our previous findings and using dietary, epigenetic, fetal biometry, anthropometric and cardiometabolic health measures from each of the participating cohorts we will examine associations between maternal DII and DASH generated by WP3 and 1) offspring adiposity (**fetal biometry,** infant and child **BMI and body composition**, and **BMI trajectories**); 2) offspring cardiometabolic health (**obesity subtypes**, in particular MHO) in all participating cohorts and 3) offspring DNA methylation. Importantly the influence of maternal dietary intervention during pregnancy on these outcomes will additionally be assessed in the PEARs RCT. Secondary aims include comparison of the influence of maternal versus paternal diet and body composition/gestational weight gain on offspring adiposity, cardiometabolic health and epigenetic patterns. **Measures available:** Main outcomes are 1) **fetal biometry** (fetal ultrasound measurements including head circumference, biparietal diameter, abdominal circumference, femur length); 2) **adiposity** (weight, height/length, BMI percentile) at birth and over the follow-up period (**BMI trajectory**) and 3) **MHO status** (as is the case for adults no standard MHO definition exists among children and adolescents. In the current project MHO characterisation will centre on the absence of MetS (or some of its cardiometabolic risk factors) among those with excess body weight based on BMI percentile for age. Additional profiling will include serum profiling of selected metabolic markers in some cohorts (EDEN). Illumina Infinium MethylationEPIC Bead Chip analysis of PEARs DNA samples will be undertaken as part of this project. **Statistical analysis:** Data will be harmonised and covariates available in each of the cohorts identified. Both cohort specific analyses and large scale meta-analyses will be conducted. Multivariable linear and logistic regression models will be used to assess associations between maternal diet (dietary quality/inflammatory potential) and offspring adiposity and cardiometabolic health outcomes. Epigenome-wide data will be analysed using linear and logistic multiple regression models in individual cohorts, adjusting for the relevant confounders, and summary results will be shared to facilitate meta-analysis between multiple cohorts using fixed effects inverse-variance weighted meta-analysis. Where a suitable genetic instrument exists, Mendelian Randomisation analyses will be undertaken to elucidate potential causal relationships. Analyses will also be informed by results from other WPs.
