# Supplementary material for: Associations of maternal dietary inflammatory potential and quality with offspring birth outcomes: An individual participant data pooled analysis of 7 European cohorts in the ALPHABET consortium
Source: PLoS Med. 2021 Jan 21;18(1):e1003491. doi: 10.1371/journal.pmed.1003491 (PMC7819611; doi:10.1371/journal.pmed.1003491)
Supplement: S1 Table — (DOCX) [file pmed.1003491.s003.docx]

**S1 Table** Characteristics of study participants according to included studies^1^

| **Cohort name** | ALPHABET overall | ALSPAC | EDEN | GEN R | Lifeways | Repro_PL | ROLO | SWS | ***P***-value |
| --- | --- | --- | --- | --- | --- | --- | --- | --- | --- |
| **Max number included** | 24861 | 11770 | 1642 | 6245 | 959 | 1164 | 573 | 2508 |  |
| Pre-pregnancy E-DII, mean ± SD | 0.1 ± 1.7 | - | 0.5 ± 1.7 | - | - | - | - | -0.2 ± 1.6 | <0.001 |
| Pregnancy E-DII, mean ± SD | 0.2 ± 1.7 | 0.6 ± 1.8 | 0.8 ± 1.6 | -0.3 ± 1.1 | 0.2 ± 1.7 | -1.1 ± 1.5 | 0.1 ± 1.7 | 0.4 ± 1.3 | <0.001 |
| Pre-pregnancy DASH, mean ± SD | 24.1 ± 4.3 | - | 24.0 ± 4.2 | - | - | - | - | 24.3 ± 4.4 | 0.046 |
| Pregnancy DASH, mean ± SD | 24.0 ± 4.2 | 24.0 ± 4.0 | 23.9 ± 4.2 | 24.0 ± 4.5 | 23.9 ± 4.6 | 24.1 ± 4.4 | 23.9 ± 4.1 | 24.2 ± 4.0 | 0.24 |
| Maternal age, y, mean ± SD | 29.5 ± 4.9 | 28.7 ± 4.8 | 29.6 ± 4.9 | 30.1 ± 5.1 | 30.1 ± 5.8 | 29.2 ± 4.2 | 32.7 ± 4.0 | 30.7 ± 3.8 | <0.001 |
| Pre-pregnancy BMI, kg/m^2^, mean ± SD | 23.3 ± 4.2 | 22.6 ± 4.1 | 23.3 ± 4.5 | 23.5 ± 3.9 | 23.9 ± 3.8 | 22.3 ± 3.6 | 26.6 ± 4.7 | 25.3 ± 4.7 | <0.001 |
| Maternal height, cm, mean ± SD | 165.0 ± 6.9 | 164.0 ± 6.5 | 163.5 ± 6.1 | 168.0 ± 7.3 | 163.7 ± 6.2 | 166.2 ± 5.8 | 166.1 ± 6.4 | 163.2 ± 6.5 | <0.001 |
| *Maternal education level* |  |  |  |  |  |  |  |  | <0.001 |
| Low | 4196 (16.9%) | 2320 (19.7%) | 103 (6.3%) | 499 (8.0%) | 164 (17.1%) | 93 (8.0%) | 5 (0.9%) | 1012 (40.4%) |  |
| Medium | 12837 (51.6%) | 7942 (67.5%) | 322 (19.6%) | 2692 (43.1%) | 510 (53.2%) | 332 (28.5%) | 125 (21.8%) | 914 (36.4%) |  |
| High | 7828 (31.5%) | 1508 (12.8%) | 1217 (74.1%) | 3054 (48.9%) | 285 (29.7%) | 739 (63.5%) | 443 (77.3%) | 582 (23.2%) |  |
| *Maternal ethnicity^3^* |  |  |  |  |  |  |  |  | <0.001 |
| European-born/White | 22358 (89.9%) | 11548 (98.1%) | 1587 (96.7%) | 4136 (66.2%) | 959 (100.0%) | 1164 (100.0%) | 562 (98.1%) | 2402 (95.8%) |  |
| Non-European-born/non-White | 2503 (10.1%) | 222 (1.9%) | 55 (3.3%) | 2109 (33.8%) | 0 (0.0%) | 0 (0.0%) | 11 (1.9%) | 106 (4.2%) |  |
| *Parity* |  |  |  |  |  |  |  |  | <0.001 |
| Nulliparous | 11824 (47.6%) | 5108 (43.4%) | 734 (44.7%) | 3629 (58.1%) | 417 (43.5%) | 675 (58.0%) | 0 (0.0%) | 1261 (50.3%) |  |
| Non-nulliparous | 13037 (52.4%) | 6662 (56.6%) | 908 (55.3%) | 2616 (41.9%) | 542 (56.5%) | 489 (42.0%) | 573 (100.0%) | 1247 (49.7%) |  |
| *Cigarette smoking during pregnancy* |  |  |  |  |  |  |  |  | <0.001 |
| Never | 14345 (57.7%) | 6098 (51.8%) | 1217 (74.1%) | 3893 (62.3%) | 407 (42.4%) | 733 (63.0%) | 554 (96.7%) | 1443 (57.5%) |  |
| Ever | 5720 (23.0%) | 2884 (24.5%) | 4 (0.2%) | 1575 (25.2%) | 282 (29.4%) | 279 (24.0%) | 0 (0.0%) | 696 (27.8%) |  |
| Current | 4796 (19.3%) | 2788 (23.7%) | 421 (25.6%) | 777 (12.4%) | 270 (28.2%) | 152 (13.1%) | 19 (3.3%) | 369 (14.7%) |  |
| *Alcohol consumption during pregnancy* |  |  |  |  |  |  |  |  | <0.001 |
| No | 12016 (48.3%) | 5157 (43.8%) | 1196 (72.8%) | 3581 (57.3%) | 284 (29.6%) | 1091 (93.7%) | 169 (29.5%) | 538 (21.5%) |  |
| Yes | 12845 (51.7%) | 6613 (56.2%) | 446 (27.2%) | 2664 (42.7%) | 675 (70.4%) | 73 (6.3%) | 404 (70.5%) | 1970 (78.5%) |  |
| Birth weight, g, mean ± SD | 3437.5 ± 543.5 | 3432.9 ± 524.6 | 3282.4 ± 503.3 | 3427.3 ± 560.8 | 3516.0 ± 568.2 | 3367.5 ± 476.9 | 4037.9 ± 468.6 | 3450.3 ± 551.4 | <0.001 |
| Birth gestational age, wk, mean ± SD | 39.6 ± 1.8 | 39.5 ± 1.7 | 39.3 ± 1.7 | 39.9 ± 1.8 | 40.0 ± 1.8 | 39.2 ± 1.4 | 40.3 ± 1.1 | 39.8 ± 1.8 | <0.001 |
| *Child sex* |  |  |  |  |  |  |  |  | 0.72 |
| Male | 12690 (51.0%) | 6035 (51.3%) | 857 (52.2%) | 3157 (50.6%) | 474 (49.4%) | 585 (50.3%) | 286 (49.9%) | 1296 (51.7%) |  |
| Female | 12171 (49.0%) | 5735 (48.7%) | 785 (47.8%) | 3088 (49.4%) | 485 (50.6%) | 579 (49.7%) | 287 (50.1%) | 1212 (48.3%) |  |

^1^Values were mean ± SD for continuous variables or n (%) for categorical variables.

^2^*P*-values were obtained from one-way ANOVA for continuous variables and chi-square test for categorical variables.

^3^For EDEN, maternal ethnicity was proxied by place of birth using the question ‘Are you born in Europe? Outside Europe?’, because specific question on ethnicity is not allowed in France.
