# Supplementary material for: Associations of maternal dietary inflammatory potential and quality with offspring birth outcomes: An individual participant data pooled analysis of 7 European cohorts in the ALPHABET consortium
Source: PLoS Med. 2021 Jan 21;18(1):e1003491. doi: 10.1371/journal.pmed.1003491 (PMC7819611; doi:10.1371/journal.pmed.1003491)
Supplement: S2 Table — (DOCX) [file pmed.1003491.s004.docx]

**S2 Table** Information on data request contacts, full cohort recruitment date, and local institutional ethical review boards for each cohort.

| **Cohort name (country)** | **Contact for data requests** | **Full cohort recruitment date (including day and month)** | **Local institutional ethical review boards** |
| --- | --- | --- | --- |
| ALSPAC (United Kingdom) | <http://www.bristol.ac.uk/alspac/researchers/> | 1 April 1991 to 31 December 1992 | ALSPAC Ethics and Law Committee and Local Research Ethics Committees |
| EDEN (France) | [etude.eden@inserm.fr](mailto:etude.eden@inserm.fr) | 27 January 2003 to 6 March 2006 | Ethics Committee of the Bicêtre Hospital |
| Generation R (The Netherlands) | [generationr@erasmusmc.nl](mailto:generationr@erasmusmc.nl) | Pregnant women with an expected delivery date between April 2002 and January 2006 | Medical Ethical Committee of the Erasmus Medical Center, Rotterdam |
| Lifeways (Republic of Ireland) | [lifeways@ucd.ie](mailto:lifeways@ucd.ie) | 2 October 2001 to 4 April 2003 | University College Dublin Research Ethics Committee and St. Vincent’s University Hospital Research Ethics Committee |
| REPRO_PL (Poland) | [impx@imp.lodz.pl](mailto:impx@imp.lodz.pl) | 18 September 2007 to 16 December 2011 | Ethical Committee of the Nofer Institute of Occupational Medicine, Łódź, Poland (Decision No. 7/2007) |
| ROLO (Republic of Ireland) | [rolostudy@gmail.com](mailto:rolostudy@gmail.com) | 1 January 2007 to 1 January 2011 | Ethics Committee of the National Maternity Hospital, Dublin, Ireland |
| SWS (United Kingdom) | [sws@mrc.soton.ac.uk](mailto:sws@mrc.soton.ac.uk) | 6 April 1998 to 17 Dec 2002 | Southampton and South West Hampshire Local Research Ethics Committee (06/Q1702/104) |
