# Supplementary material for: Associations of maternal dietary inflammatory potential and quality with offspring birth outcomes: An individual participant data pooled analysis of 7 European cohorts in the ALPHABET consortium
Source: PLoS Med. 2021 Jan 21;18(1):e1003491. doi: 10.1371/journal.pmed.1003491 (PMC7819611; doi:10.1371/journal.pmed.1003491)
Supplement: S3 Table — (DOCX) [file pmed.1003491.s005.docx]

**S3 Table** Food Frequency Questionnaires and Diet scores in the ALPHABET consortium.

| **Cohort** | **ALSPAC** | **EDEN** | **Generation R** | **Lifeways** | **REPRO_PL** | **ROLO** | **SWS** | **ALPHABET ^1^** |
| --- | --- | --- | --- | --- | --- | --- | --- | --- |
| **FFQ** | | | | | | | | |
| FFQ total of food items | 43 | 137 | 293 | 158 | 66 | 158 | 104 | 137.0 |
| FFQ total of food items without alcohol | 43 | 130 | 283 | 154 | 62 | 154 | 99 | 132.1 |
| FFQ response categories | 5 | 7 | 9 | 9 | 6 | 9 | ≥8 | 7.6 |
| **E-DII** | | | | | | | | |
| Number of dietary parameters used | 28 | 25 | 20 | 28 | 28 | 28 | 24 | 25.9 |
| % ^2^ dietary parameters used/45 dietary parameters possible | 63.6% | 56.8% | 45.5% | 63.6% | 63.6% | 63.6% | 54.5% | 58.9% |
| **DASH** | | | | | | | | |
| Total of food items selected for the DASH | 34 | 65 | 136 | 85 | 36 | 85 | 58 | 71.3 |
| % ^2^ items selected/total food items without alcohol | 79.1% | 50.0% | 48.1% | 55.2% | 58.1% | 55.2% | 58.6% | 57.8% |
| **Food components with higher intakes recommended** | | | | | | | | |
| Total grains | 7 | 7 | 20 | 14 | 5 | 14 | 8 | 10.7 |
| Vegetables (excluding potatoes and condiments) | 5 | 16 | 33 | 24 | 12 | 24 | 16 | 18.6 |
| Fruits | 3 | 12 | 20 | 13 | 10 | 13 | 12 | 11.9 |
| Non-full-fat dairy products | 3 | 6 | 18 ^3^ | 7 | 2 | 7 | 5 | 6.9 |
| Nuts, seeds, legumes | 7 | 4 | 14 | 5 | 2 | 5 | 2 | 5.6 |
| **Food components with lower intakes recommended** | | | | | | | | |
| Red and Processed meat | 4 | 12 | 20 | 17 | 4 | 17 | 10 | 12.0 |
| Sugar-sweetened beverages, sweets, and added sugars | 5 | 8 | 11 | 5 | 1 | 5 | 5 | 5.7 |
| Sodium | Available in grams/day | Available in grams/day | Available in grams/day | Available in grams/day | Available in grams/day | Available in grams/day | Available in grams/day | Available in grams/day |

FFQ: Food frequency questionnaire. E-DII: Energy-adjusted Dietary Inflammatory Index. DASH: Dietary Approaches to Stop Hypertension.

^1^Mean values (rounded to one decimal point) in the ALPHABET consortium.

^2^Percentage (rounded to one decimal point).

^3^By combining items on foods and items on types of milk consumed.
