# Supplementary material for: Associations of maternal dietary inflammatory potential and quality with offspring birth outcomes: An individual participant data pooled analysis of 7 European cohorts in the ALPHABET consortium
Source: PLoS Med. 2021 Jan 21;18(1):e1003491. doi: 10.1371/journal.pmed.1003491 (PMC7819611; doi:10.1371/journal.pmed.1003491)
Supplement: S4 Table — (DOCX) [file pmed.1003491.s006.docx]

**S4 Table** Proportions of infants with adverse birth outcomes

| **Binary outcomes** | **n (%)** |
| --- | --- |
| Low birth weight |  |
| No | 23060 (96.1%) |
| Yes | 931 (3.9%) |
| Small-for-gestational age |  |
| No | 22578 (94.9%) |
| Yes | 1212 (5.1%) |
| Macrosomia |  |
| No | 20612 (85.9%) |
| Yes | 3379 (14.1%) |
| Large-for-gestational age |  |
| No | 18561 (78.0%) |
| Yes | 5229 (22.0%) |
| Preterm birth |  |
| No | 22992 (95.4%) |
| Yes | 1108 (4.6%) |
| Of which spontaneous | 926 (83.6%) |
| Post-term birth |  |
| No | 22545 (93.5%) |
| Yes | 1555 (6.5%) |
| Of which spontaneous | 1163 (74.8%) |
