# Supplementary material for: Associations of maternal dietary inflammatory potential and quality with offspring birth outcomes: An individual participant data pooled analysis of 7 European cohorts in the ALPHABET consortium
Source: PLoS Med. 2021 Jan 21;18(1):e1003491. doi: 10.1371/journal.pmed.1003491 (PMC7819611; doi:10.1371/journal.pmed.1003491)
Supplement: S5 Table — (DOCX) [file pmed.1003491.s007.docx]

**S5 Table** Sensitivity analysis for continuous outcomes- complete case analysis

|  | Primary outcomes | | | |  | Secondary outcomes | | | | | | | |
| --- | --- | --- | --- | --- | --- | --- | --- | --- | --- | --- | --- | --- | --- |
|  | Birthweight, g |  | Gestational age, wk |  |  | Birth length, cm |  | Head circumference, cm |  | Abdominal circumference, cm |  | Sum of skinfold thickness, mm |  |
|  | β (95%CI) | *I^2^ (%)* | β (95%CI) | *I^2^ (%)* |  | β (95%CI) | *I^2^ (%)* | β (95%CI) | *I^2^ (%)* | β (95%CI) | *I^2^ (%)* | β (95%CI) | *I^2^ (%)* |
| **E-DII** |  |  |  |  |  |  |  |  |  |  |  |  |  |
| *Pre* | -19.9 (-36.3, -3.6)* | 0 | -0.03 (-0.09, 0.03) | 7 |  | -0.08 (-0.15, -0.01)* | 0 | -0.05 (-0.13, 0.04) | 67 | -0.01 (-0.09, 0.08) | - | -0.04 (-0.09, 0.02) | 0 |
| Np/Nc | 3973/2 |  | 3990/2 |  |  | 3823/2 |  | 3851/2 |  | 2406/1 |  | 3791/2 |  |
| *Preg* | -12.1 (-27.4, 3.2) | 71* | -0.02 (-0.06, 0.03) | 68* |  | -0.04 (-0.09, 0.01) | 23 | -0.03 (-0.07, 0.01) | 58* | 0.02 (-0.07, 0.11) | 0 | -0.02 (-0.10, 0.05) | 21 |
| Np/Nc | 19620/7 |  | 19678/7 |  |  | 15794/7 |  | 15256/7 |  | 2020/2 |  | 3388/3 |  |
| *Early* | -9.0 (-34.1, 16.1) | 77* | -0.02 (-0.09, 0.05) | 69* |  | -0.04 (-0.11, 0.04) | 39 | -0.02 (-0.09, 0.05) | 69* | 0.002 (-0.11, 0.11) | 10 | -0.12 (-0.35, 0.11) | 45 |
| Np/Nc | 9317/5 |  | 9284/5 |  |  | 7309/5 |  | 6698/5 |  | 2118/2 |  | 2073/2 |  |
| *Late* | -14.9 (-28.4, -1.3)* | 46 | -0.01 (-0.07, 0.06) | 73* |  | -0.04 (-0.08, 0.004) | 0 | -0.03 (-0.06, -0.01)* | 0 | -0.02 (-0.10, 0.07) | - | 0.01 (-0.05, 0.07) | 0 |
| Np/Nc | 12794/3 |  | 12902/3 |  |  | 10868/3 |  | 10969/3 |  | 2309/1 |  | 3708/2 |  |
|  |  |  |  |  |  |  |  |  |  |  |  |  |  |
| **DASH** |  |  |  |  |  |  |  |  |  |  |  |  |  |
| *Pre* | 18.3 (-4.5, 41.1) | 45 | 0.01 (-0.05, 0.06) | 0 |  | 0.08 (0.01, 0.15)* | 0 | 0.01 (-0.04, 0.06) | 0 | 0.10 (0.01, 0.18)* | - | 0.06 (-0.02, 0.14) | 46 |
| Np/Nc | 3973/2 |  | 3990/2 |  |  | 3823/2 |  | 3851/2 |  | 2406/1 |  | 3791/2 |  |
| *Preg* | 17.8 (9.0, 26.6)*** | 20 | 0.02 (-0.00, 0.04) | 0 |  | 0.05 (0.01, 0.08)* | 0 | 0.03 (0.01, 0.06)** | 0 | -0.04 (-0.34, 0.26) | 73 | 0.07 (0.004, 0.13)* | 0 |
| Np/Nc | 19619/7 |  | 19677/7 |  |  | 15793/7 |  | 15256/7 |  | 2020/2 |  | 3388/3 |  |
| *Early* | 16.0 (-0.2, 32.2) | 43 | 0.02 (-0.01, 0.06) | 0 |  | 0.06 (0.002, 0.11)* | 0 | 0.03 (-0.01, 0.06) | 0 | -0.04 (-0.33, 0.25) | 71 | 0.12 (0.03, 0.20)** | 0 |
| Np/Nc | 9316/5 |  | 9283/5 |  |  | 7308/5 |  | 6698/5 |  | 2118/2 |  | 2073/2 |  |
| *Late* | 18.4 (10.0, 26.7)*** | 0 | 0.02 (-0.003, 0.05) | 0 |  | 0.06 (0.003, 0.12)* | 34 | 0.04 (0.01, 0.07)** | 0 | 0.11 (0.02, 0.20)* | - | 0.04 (-0.02, 0.10) | 0 |
| Np/Nc | 12793/3 |  | 12901/3 |  |  | 10867/3 |  | 10968/3 |  | 2308/1 |  | 3707/2 |  |

Values are adjusted pooled effect estimates [β (95% CI)] expressed for a 1-SD increment in dietary scores, heterogeneity measure (*I*^2^), and number of participants and studies included (Np/Nc) across different outcomes and conception periods, as labelled. Effect estimates were adjusted for maternal education, pre-pregnancy BMI, maternal height, parity, energy intake (for DASH), cigarette smoking and alcohol consumption during pregnancy, and child sex.

E-DII, energy-adjusted Dietary Inflammatory Index; DASH, Dietary Approaches to Stop Hypertension; *I*^2^, *I*-squared; Pre, pre-pregnancy; Preg, pregnancy; Early, early pregnancy; Late, late pregnancy; Np, number of participants included; Nc, number of cohorts included.

**P*<0.05, ***P*<0.01, ****P*<0.001
