# Supplementary material for: Associations of maternal dietary inflammatory potential and quality with offspring birth outcomes: An individual participant data pooled analysis of 7 European cohorts in the ALPHABET consortium
Source: PLoS Med. 2021 Jan 21;18(1):e1003491. doi: 10.1371/journal.pmed.1003491 (PMC7819611; doi:10.1371/journal.pmed.1003491)
Supplement: S6 Table — (DOCX) [file pmed.1003491.s008.docx]

**S6 Table** Sensitivity analysis for binary outcomes- complete case analysis

|  | Low birth weight |  | SGA |  | Macrosomia |  | LGA |  | Preterm birth |  | Post-term birth |  |
| --- | --- | --- | --- | --- | --- | --- | --- | --- | --- | --- | --- | --- |
|  | OR (95% CI) | *I^2^ (%)* | OR (95% CI) | *I^2^ (%)* | OR (95% CI) | *I^2^ (%)* | OR (95% CI) | *I^2^ (%)* | OR (95% CI) | *I^2^ (%)* | OR (95% CI) | *I^2^ (%)* |
| **E-DII** |  |  |  |  |  |  |  |  |  |  |  |  |
| Pre | 1.18 (1.001, 1.38)* | 0 | 1.14 (0.99, 1.33) | 0 | 0.99 (0.88, 1.10) | 0 | 0.97 (0.89, 1.05) | 0 | 1.05 (0.91, 1.21) | 0 | 1.07 (0.90, 1.28) | 0 |
| Np/Nc | 3973/2 |  | 3973/2 |  | 3923/2 |  | 3973/2 |  | 3990/2 |  | 3990/2 |  |
| Preg | 1.13 (1.00, 1.27)* | 27 | 1.18 (1.10, 1.26)*** | 0 | 0.96 (0.88, 1.06) | 67** | 0.99 (0.92, 1.06) | 65** | 1.01 (0.89, 1.15) | 26 | 0.98 (0.91, 1.05) | 10 |
| Np/Nc | 19215/7 |  | 19065/7 |  | 19569/7 |  | 19470/7 |  | 10503/6 |  | 19582/7 |  |
| Early | 1.15 (1.03, 1.29)* | 0 | 1.15 (1.03, 1.27)* | 0 | 1.01 (0.88, 1.16) | 74** | 1.02 (0.91, 1.15) | 74** | 1.08 (0.98, 1.20) | 0 | 1.00 (0.91, 1.10) | 0 |
| Np/Nc | 8912/5 |  | 8804/5 |  | 9317/5 |  | 9209/5 |  | 9146/5 |  | 9188/5 |  |
| Late | 1.07 (0.84, 1.36) | 69* | 1.19 (1.09, 1.30)*** | 0 | 0.93 (0.84, 1.04) | 56 | 0.95 (0.91, 1.00)* | 2 | 0.95 (0.69, 1.31) | 74 | 1.07 (0.87, 1.31) | 68* |
| Np/Nc | 12794/3 |  | 12752/3 |  | 12743/3 |  | 12752/3 |  | 3865/2 |  | 12902/3 |  |
|  |  |  |  |  |  |  |  |  |  |  |  |  |
| **DASH** |  |  |  |  |  |  |  |  |  |  |  |  |
| Pre | 0.87 (0.62, 1.24) | 78* | 0.86 (0.70, 1.05) | 43 | 1.04 (0.93, 1.17) | 0 | 1.06 (0.93, 1.20) | 44 | 0.94 (0.81, 1.09) | 0 | 0.93 (0.73, 1.18) | 30 |
| Np/Nc | 3973/2 |  | 3973/2 |  | 3923/2 |  | 3973/2 |  | 3990/2 |  | 3990/2 |  |
| Preg | 0.87 (0.79, 0.96)** | 0 | 0.87 (0.81, 0.93)*** | 0 | 1.03 (0.97, 1.10) | 34 | 1.06 (1.001, 1.12)* | 34 | 0.97 (0.87, 1.09) | 11 | 1.00 (0.92, 1.08) | 15 |
| Np/Nc | 19214/7 |  | 19064/7 |  | 19568/7 |  | 19469/7 |  | 10502/6 |  | 19581/7 |  |
| Early | 0.88 (0.78, 0.99)* | 0 | 0.84 (0.75, 0.94)** | 0 | 1.00 (0.92, 1.09) | 36 | 1.04 (0.96, 1.13) | 43 | 0.99 (0.87, 1.12) | 14 | 0.98 (0.89, 1.08) | 0 |
| Np/Nc | 8911/5 |  | 8803/5 |  | 9316/5 |  | 9208/5 |  | 9145/5 |  | 9187/5 |  |
| Late | 0.84 (0.74, 0.96)* | 0 | 0.86 (0.78, 0.96)** | 16 | 1.07 (1.00, 1.14)* | 11 | 1.09 (1.02, 1.16)* | 25 | 0.87 (0.72, 1.03) | 0 | 0.93 (0.77, 1.13) | 61 |
| Np/Nc | 12793/3 |  | 12751/3 |  | 12742/3 |  | 12751/3 |  | 3864/2 |  | 12901/3 |  |

Values are adjusted pooled effect estimates [OR (95% CI)] expressed for a 1-SD increment in dietary scores, heterogeneity measure (*I*^2^), and number of participants and studies included (Np/Nc) across different outcomes and conception periods, as labelled. Effect estimates were adjusted for maternal education, pre-pregnancy BMI, maternal height, parity, energy intake (for DASH), cigarette smoking and alcohol consumption during pregnancy, and child sex.

E-DII, energy-adjusted Dietary Inflammatory Index; DASH, Dietary Approaches to Stop Hypertension; *I*^2^, *I*-squared; SGA, small-for-gestational-age; LGA, large-for-gestational-age; Pre, pre-pregnancy; Preg, pregnancy; Early, early pregnancy; Late, late pregnancy; Np, number of participants included; Nc, number of cohorts included.

**P*<0.05, ***P*<0.01, ****P*<0.001
