# Supplementary material for: Associations of maternal dietary inflammatory potential and quality with offspring birth outcomes: An individual participant data pooled analysis of 7 European cohorts in the ALPHABET consortium
Source: PLoS Med. 2021 Jan 21;18(1):e1003491. doi: 10.1371/journal.pmed.1003491 (PMC7819611; doi:10.1371/journal.pmed.1003491)
Supplement: S7 Table — (DOCX) [file pmed.1003491.s009.docx]

**S7 Table** Sensitivity analysis for continuous outcomes- excluding all non-European-born/non-White participants

|  | Primary outcomes | | | |  | Secondary outcomes | | | | | | | |
| --- | --- | --- | --- | --- | --- | --- | --- | --- | --- | --- | --- | --- | --- |
|  | Birthweight, g |  | Gestational age, wk |  |  | Birth length, cm |  | Head circumference, cm |  | Abdominal circumference, cm |  | Sum of skinfold thickness, mm |  |
|  | β (95%CI) | *I^2^ (%)* | β (95%CI) | *I^2^ (%)* |  | β (95%CI) | *I^2^ (%)* | β (95%CI) | *I^2^ (%)* | β (95%CI) | *I^2^ (%)* | β (95%CI) | *I^2^ (%)* |
| **E-DII** |  |  |  |  |  |  |  |  |  |  |  |  |  |
| *Pre* | -17.2 (-33.5, -0.9)* | 0 | -0.01 (-0.07, 0.05) | 0 |  | -0.08 (-0.15, -0.01)* | 0 | -0.04 (-0.12, 0.04) | 64 | -0.01 (-0.10, 0.08) | - | -0.02 (-0.08, 0.03) | 0 |
| Np/Nc | 3959/2 |  | 3977/2 |  |  | 3812/2 |  | 3840/2 |  | 2305/1 |  | 3772/2 |  |
| *Preg* | -13.0 (-26.5, 0.6) | 62* | -0.01 (-0.05, 0.03) | 59* |  | -0.05 (-0.09, -0.02)* | 0 | -0.03 (-0.07, 0.01) | 43 | 0.10 (-0.13, 0.32) | 60 | -0.01 (-0.07, 0.05) | 0 |
| Np/Nc | 21549/7 |  | 21645/7 |  |  | 17372/7 |  | 16932/7 |  | 1964/2 |  | 3413/3 |  |
| *Early* | -9.4 (-30.0, 11.1) | 65* | -0.02 (-0.07, 0.04) | 46 |  | -0.04 (-0.09, 0.02) | 1 | -0.02 (-0.08, 0.04) | 57 | 0.09 (-0.17, 0.34) | 67 | -0.06 (-0.14, 0.03) | 0 |
| Np/Nc | 8690/5 |  | 8641/5 |  |  | 7022/5 |  | 6463/5 |  | 2060/2 |  | 2013/2 |  |
| *Late* | -16.2 (-30.8, -1.5) | 53 | 0.01 (-0.07, 0.08) | 77* |  | -0.06 (-0.10, -0.02)* | 0 | -0.04 (-0.06, -0.01)** | 0 | -0.03 (-0.12, 0.05) | - | 0.01 (-0.05, 0.06) | 0 |
| Np/Nc | 15244/3 |  | 15406/3 |  |  | 12633/3 |  | 12778/3 |  | 2210/1 |  | 3692/2 |  |
|  |  |  |  |  |  |  |  |  |  |  |  |  |  |
| **DASH** |  |  |  |  |  |  |  |  |  |  |  |  |  |
| *Pre* | 19.1 (-7.6, 45.8) | 60 | -0.01 (-0.08, 0.06) | 35 |  | 0.08 (0.01, 0.15)* | 0 | 0.02 (-0.03, 0.06) | 0 | 0.11 (0.02, 0.20) | - | 0.07 (-0.01, 0.15) | 46 |
| Np/Nc | 3959/2 |  | 3977/2 |  |  | 3812/2 |  | 3840/2 |  | 2305/1 |  | 3772/2 |  |
| *Preg* | 18.3 (5.8, 30.9)** | 54* | 0.02 (-0.02, 0.05) | 43 |  | 0.05 (0.01, 0.09)** | 0 | 0.04 (0.01, 0.06)** | 0 | -0.01 (-0.26, 0.24) | 63 | 0.06 (-0.03, 0.14) | 36 |
| Np/Nc | 21547/7 |  | 21644/7 |  |  | 17371/7 |  | 16932/7 |  | 1963/2 |  | 3412/3 |  |
| *Early* | 22.0 (3.5, 40.5)* | 56 | 0.03 (-0.03, 0.08) | 46 |  | 0.08 (0.02, 0.14)** | 0 | 0.03 (-0.01, 0.07) | 0 | -0.01 (-0.26, 0.23) | 0 | 0.12 (0.03, 0.20)** | 0 |
| Np/Nc | 8688/5 |  | 8640/5 |  |  | 7021/5 |  | 6463/5 |  | 2059/2 |  | 2012/2 |  |
| *Late* | 15.3 (4.7, 25.8)** | 20 | 0.02 (-0.03, 0.06) | 39 |  | 0.06 (-0.01, 0.12) | 43 | 0.04 (0.01, 0.06)** | 0 | 0.10 (0.01, 0.19)* | - | 0.03 (-0.03, 0.09) | 0 |
| Np/Nc | 15243/3 |  | 15405/3 |  |  | 12632/3 |  | 12777/3 |  | 2209/1 |  | 3691/2 |  |

Values are adjusted pooled effect estimates [β (95% CI)] expressed for a 1-SD increment in dietary scores, heterogeneity measure (*I*^2^), and number of participants and studies included (Np/Nc) across different outcomes and conception periods, as labelled. Effect estimates were adjusted for maternal education, pre-pregnancy BMI, maternal height, parity, energy intake (for DASH), cigarette smoking and alcohol consumption during pregnancy, and child sex.

E-DII, energy-adjusted Dietary Inflammatory Index; DASH, Dietary Approaches to Stop Hypertension; *I*^2^, *I*-squared; Pre, pre-pregnancy; Preg, pregnancy; Early, early pregnancy; Late, late pregnancy; Np, number of participants included; Nc, number of cohorts included.

**P*<0.05, ***P*<0.01, ****P*<0.001
