# Supplementary material for: Associations of maternal dietary inflammatory potential and quality with offspring birth outcomes: An individual participant data pooled analysis of 7 European cohorts in the ALPHABET consortium
Source: PLoS Med. 2021 Jan 21;18(1):e1003491. doi: 10.1371/journal.pmed.1003491 (PMC7819611; doi:10.1371/journal.pmed.1003491)
Supplement: S8 Table — (DOCX) [file pmed.1003491.s010.docx]

**S8 Table** Sensitivity analysis for binary outcomes- excluding all non-European-born/non-White participants

|  | Low birth weight |  | SGA |  | Macrosomia |  | LGA |  | Preterm birth |  | Post-term birth |  |
| --- | --- | --- | --- | --- | --- | --- | --- | --- | --- | --- | --- | --- |
|  | OR (95% CI) | *I^2^ (%)* | OR (95% CI) | *I^2^ (%)* | OR (95% CI) | *I^2^ (%)* | OR (95% CI) | *I^2^ (%)* | OR (95% CI) | *I^2^ (%)* | OR (95% CI) | *I^2^ (%)* |
| **E-DII** |  |  |  |  |  |  |  |  |  |  |  |  |
| Pre | 1.17 (0.99, 1.37) | 0 | 1.12 (0.97, 1.31) | 0 | 0.98 (0.88, 1.09) | 0 | 0.97 (0.89, 1.05) | 0 | 1.03 (0.89, 1.18) | 0 | 1.13 (0.95, 1.36) | 0 |
| Np/Nc | 3959/2 |  | 3959/2 |  | 3959/2 |  | 3959/2 |  | 3977/2 |  | 3977/2 |  |
| Preg | 1.14 (1.02, 1.27)* | 30 | 1.21 (1.13, 1.30)*** | 0 | 0.97 (0.90, 1.04) | 43 | 0.99 (0.93, 1.06) | 59* | 1.01 (0.93, 1.10) | 14 | 1.01 (0.92, 1.11) | 28 |
| Np/Nc | 21138/7 |  | 20946/7 |  | 21549/7 |  | 21356/7 |  | 21508/7 |  | 21553/7 |  |
| Early | 1.18 (1.05, 1.33)** | 0 | 1.25 (1.1, 1.40)*** | 0 | 1.01 (0.95, 1.07) | 0 | 1.03 (0.94, 1.13) | 60* | 1.06 (0.95, 1.17) | 0 | 1.02 (0.93, 1.13) | 0 |
| Np/Nc | 8279/5 |  | 8138/5 |  | 8690/5 |  | 8548/5 |  | 8504/5 |  | 8549/5 |  |
| Late | 1.06 (0.85, 1.33) | 72* | 1.18 (1.09, 1.28)*** | 0 | 0.93 (0.84, 1.03) | 52 | 0.95 (0.90, 0.99)* | 1 | 0.98 (0.82, 1.17) | 67 | 1.09 (0.88, 1.37) | 72* |
| Np/Nc | 15244/3 |  | 15193/3 |  | 15244/3 |  | 15193/3 |  | 15406/3 |  | 15406/3 |  |
|  |  |  |  |  |  |  |  |  |  |  |  |  |
| **DASH** |  |  |  |  |  |  |  |  |  |  |  |  |
| Pre | 0.87 (0.57, 1.34) | 85* | 0.86 (0.73, 1.02) | 15 | 1.06 (0.95, 1.18) | 0 | 1.06 (0.93, 1.22) | 53 | 0.95 (0.82, 1.10) | 0 | 0.89 (0.69, 1.14) | 36 |
| Np/Nc | 3959/2 |  | 3959/2 |  | 3959/2 |  | 3959/2 |  | 3977/2 |  | 3977/2 |  |
| Preg | 0.87 (0.80, 0.96)** | 12 | 0.86 (0.77, 0.96)** | 40 | 1.03 (0.97, 1.08) | 19 | 1.06 (0.99, 1.12) | 49 | 0.96 (0.87, 1.05) | 22 | 0.97 (0.87, 1.09) | 45 |
| Np/Nc | 21137/7 |  | 20945/7 |  | 21547/7 |  | 21355/7 |  | 21507/7 |  | 21552/7 |  |
| Early | 0.80 (0.71, 0.90)*** | 0 | 0.82 (0.70, 0.96)* | 28 | 1.03 (0.97, 1.10) | 0 | 1.05 (0.97, 1.14) | 44 | 0.97 (0.82, 1.15) | 43 | 0.96 (0.84, 1.11) | 28 |
| Np/Nc | 8278/5 |  | 8137/5 |  | 8688/5 |  | 8547/5 |  | 8503/5 |  | 8548/5 |  |
| Late | 0.91 (0.83, 0.998)* | 0 | 0.90 (0.82, 0.98)* | 9 | 1.07 (0.96, 1.19) | 55 | 1.09 (0.98, 1.22) | 68 | 0.94 (0.86, 1.03) | 9 | 0.90 (0.72, 1.13) | 73* |
| Np/Nc | 15243/3 |  | 15192/3 |  | 15243/3 |  | 15192/3 |  | 15405/3 |  | 15405/3 |  |

Values are adjusted pooled effect estimates [OR (95% CI)] expressed for a 1-SD increment in dietary scores, heterogeneity measure (*I*^2^), and number of participants and studies included (Np/Nc) across different outcomes and conception periods, as labelled. Effect estimates were adjusted for maternal education, pre-pregnancy BMI, maternal height, parity, energy intake (for DASH), cigarette smoking and alcohol consumption during pregnancy, and child sex.

E-DII, energy-adjusted Dietary Inflammatory Index; DASH, Dietary Approaches to Stop Hypertension; *I*^2^, *I*-squared; SGA, small-for-gestational-age; LGA, large-for-gestational-age; Pre, pre-pregnancy; Preg, pregnancy; Early, early pregnancy; Late, late pregnancy; Np, number of participants included; Nc, number of cohorts included.

**P*<0.05, ***P*<0.01, ****P*<0.001
