# Supplementary material for: Associations of maternal dietary inflammatory potential and quality with offspring birth outcomes: An individual participant data pooled analysis of 7 European cohorts in the ALPHABET consortium
Source: PLoS Med. 2021 Jan 21;18(1):e1003491. doi: 10.1371/journal.pmed.1003491 (PMC7819611; doi:10.1371/journal.pmed.1003491)
Supplement: S9 Table — (DOCX) [file pmed.1003491.s011.docx]

**S9 Table** Sensitivity analysis for continuous outcomes- excluding participants with pregnancy complications

|  | Primary outcomes | | | |  | Secondary outcomes | | | | | | | |
| --- | --- | --- | --- | --- | --- | --- | --- | --- | --- | --- | --- | --- | --- |
|  | Birthweight, g |  | Gestational age, wk |  |  | Birth length, cm |  | Head circumference, cm |  | Abdominal circumference, cm |  | Sum of skinfold thickness, mm |  |
|  | β (95%CI) | *I^2^ (%)* | β (95%CI) | *I^2^ (%)* |  | β (95%CI) | *I^2^ (%)* | β (95%CI) | *I^2^ (%)* | β (95%CI) | *I^2^ (%)* | β (95%CI) | *I^2^ (%)* |
| **E-DII** |  |  |  |  |  |  |  |  |  |  |  |  |  |
| *Pre* | -15.1 (-31.1, 0.8) | 0 | 0.003 (-0.05, 0.06) | 0 |  | -0.05 (-0.11, 0.02) | 0 | -0.03 (-0.08, 0.03) | 28 | 0.01 (-0.08, 0.09) | - | -0.01 (-0.08, 0.06) | 32 |
| Np/Nc | 3776/2 |  | 3793/2 |  |  | 3644/2 |  | 3671/2 |  | 2251/1 |  | 3615/2 |  |
| *Preg* | -13.7 (-28.8, 1.4) | 72** | -0.01 (-0.06, 0.04) | 72** |  | -0.05 (-0.11, -0.001)* | 39 | -0.04 (-0.07, 0.001) | 46 | 0.05 (-0.08, 0.19) | 21 | -0.00 (-0.10, 0.10) | 51 |
| Np/Nc | 21486/7 |  | 21574/7 |  |  | 16961/7 |  | 16358/7 |  | 1909/2 |  | 3253/3 |  |
| *Early* | -14.0 (-37.0, 9.1) | 75** | -0.03 (-0.09, 0.04) | 68* |  | -0.06 (-0.15, 0.03) | 55 | -0.03 (-0.10, 0.04) | 65* | 0.05 (-0.10, 0.21) | 31 | -0.12 (-0.36, 0.13) | 51 |
| Np/Nc | 10361/5 |  | 10325/5 |  |  | 8031/5 |  | 7326/5 |  | 1994/2 |  | 1950/2 |  |
| *Late* | -12.0 (-26.1, 2.2) | 50 | 0.02 (-0.05, 0.08) | 72* |  | -0.04 (-0.08, 0.001) | 0 | -0.03 (-0.06, -0.004)* | 0 | -0.02 (-0.10, 0.07) | - | 0.04 (-0.02, 0.09) | 0 |
| Np/Nc | 13448/3 |  | 13588/3 |  |  | 11160/3 |  | 11287/3 |  | 2167/1 |  | 3545/2 |  |
|  |  |  |  |  |  |  |  |  |  |  |  |  |  |
| **DASH** |  |  |  |  |  |  |  |  |  |  |  |  |  |
| *Pre* | 15.5 (-2.5, 33.4) | 16 | -0.02 (-0.08, 0.05) | 32 |  | 0.07 (-0.004, 0.13) | 0 | 0.01 (-0.03, 0.06) | 0 | 0.08 (-0.01, 0.17) | - | 0.06 (-0.01, 0.12) | 22 |
| Np/Nc | 3776/2 |  | 3793/2 |  |  | 3644/2 |  | 3671/2 |  | 2251/1 |  | 3615/2 |  |
| *Preg* | 16.8 (2.4, 31.2)* | 68** | 0.01 (-0.03, 0.05) | 50 |  | 0.05 (0.003, 0.10)* | 28 | 0.02 (0.00, 0.05)* | 0 | -0.002 (-0.20, 0.20) | 47 | 0.04 (-0.06, 0.14) | 46 |
| Np/Nc | 21484/7 |  | 21573/7 |  |  | 16960/7 |  | 16358/7 |  | 1908/2 |  | 3252/3 |  |
| *Early* | 20.8 (3.0, 38.6)* | 56 | 0.02 (-0.02, 0.07) | 38 |  | 0.08 (0.03, 0.13)** | 0 | 0.03 (-0.002, 0.07) | 0 | -0.01 (-0.18, 0.17) | 36 | 0.10 (0.01, 0.18) | 0 |
| Np/Nc | 10359/5 |  | 10324/5 |  |  | 8030/5 |  | 7326/5 |  | 1993/2 |  | 1949/2 |  |
| *Late* | 14.0 (-1.9, 30.0) | 57 | 0.004 (-0.05, 0.05) | 50 |  | 0.04 (-0.04, 0.12) | 61 | 0.02 (-0.01, 0.06) | 32 | 0.10 (0.01, 0.19)* | - | 0.02 (-0.07, 0.11) | 50 |
| Np/Nc | 13447/3 |  | 13587/3 |  |  | 11159/3 |  | 11286/3 |  | 2166/1 |  | 3544/2 |  |

Values are adjusted pooled effect estimates [β (95% CI)] expressed for a 1-SD increment in dietary scores, heterogeneity measure (*I*^2^), and number of participants and studies included (Np/Nc) across different outcomes and conception periods, as labelled. Effect estimates were adjusted for maternal education, pre-pregnancy BMI, ethnicity, maternal height, parity, energy intake (for DASH), cigarette smoking and alcohol consumption during pregnancy, and child sex.

E-DII, energy-adjusted Dietary Inflammatory Index; DASH, Dietary Approaches to Stop Hypertension; *I*^2^, *I*-squared; Pre, pre-pregnancy; Preg, pregnancy; Early, early pregnancy; Late, late pregnancy; Np, number of participants included; Nc, number of cohorts included.

**P*<0.05, ***P*<0.01, ****P*<0.001
