# Supplementary material for: Associations of maternal dietary inflammatory potential and quality with offspring birth outcomes: An individual participant data pooled analysis of 7 European cohorts in the ALPHABET consortium
Source: PLoS Med. 2021 Jan 21;18(1):e1003491. doi: 10.1371/journal.pmed.1003491 (PMC7819611; doi:10.1371/journal.pmed.1003491)
Supplement: S10 Table — (DOCX) [file pmed.1003491.s012.docx]

**S10 Table** Sensitivity analysis for binary outcomes- excluding participants with pregnancy complications

|  | Low birth weight |  | SGA |  | Macrosomia |  | LGA |  | Preterm birth |  | Post-term birth |  |
| --- | --- | --- | --- | --- | --- | --- | --- | --- | --- | --- | --- | --- |
|  | OR (95% CI) | *I^2^ (%)* | OR (95% CI) | *I^2^ (%)* | OR (95% CI) | *I^2^ (%)* | OR (95% CI) | *I^2^ (%)* | OR (95% CI) | *I^2^ (%)* | OR (95% CI) | *I^2^ (%)* |
| **E-DII** |  |  |  |  |  |  |  |  |  |  |  |  |
| Pre | 1.17 (0.97, 1.40) | 0 | 1.17 (1.003, 1.37)* | 0 | 0.98 (0.88, 1.10) | 0 | 0.97 (0.88, 1.06) | 0 | 1.001 (0.85, 1.17) | 0 | 1.14 (0.95, 1.38) | 0 |
| Np/Nc | 3776/2 |  | 3776/2 |  | 3727/2 |  | 3776/2 |  | 3793/2 |  | 3793/2 |  |
| Preg | 1.12 (0.98, 1.27) | 45 | 1.18 (1.10, 1.26)*** | 0 | 0.94 (0.88, 0.99)* | 30 | 0.98 (0.91, 1.05) | 61* | 1.01 (0.90, 1.14) | 50 | 0.99 (0.92, 1.07) | 12 |
| Np/Nc | 21064/7 |  | 20878/7 |  | 21436/7 |  | 21299/7 |  | 21426/7 |  | 21472/7 |  |
| Early | 1.21 (1.09, 1.35)** | 0 | 1.18 (1.07, 1.31)** | 0 | 0.98 (0.89, 1.07) | 46 | 1.01 (0.91, 1.13) | 71** | 1.11 (1.01, 1.22)* | 0 | 1.01 (0.92, 1.10) | 0 |
| Np/Nc | 9939/5 |  | 9794/5 |  | 10361/5 |  | 10215/5 |  | 10177/5 |  | 10223/5 |  |
| Late | 1.01 (0.79, 1.28) | 71* | 1.17 (1.07, 1.27)*** | 0 | 0.93 (0.85, 1.01) | 32 | 0.95 (0.89, 1.01) | 21 | 0.94 (0.76, 1.16) | 71* | 1.08 (0.88, 1.32) | 66 |
| Np/Nc | 13448/3 |  | 13407/3 |  | 13398/3 |  | 13407/3 |  | 13588/3 |  | 13588/3 |  |
|  |  |  |  |  |  |  |  |  |  |  |  |  |
| **DASH** |  |  |  |  |  |  |  |  |  |  |  |  |
| Pre | 0.88 (0.58, 1.34) | 80* | 0.83 (0.71, 0.97)* | 0 | 1.04 (0.92, 1.16) | 0 | 1.04 (0.89, 1.21) | 58 | 0.97 (0.82, 1.14) | 0 | 0.88 (0.68, 1.14) | 33 |
| Np/Nc | 3776/2 |  | 3776/2 |  | 3727/2 |  | 3776/2 |  | 3793/2 |  | 3793/2 |  |
| Preg | 0.87 (0.74, 1.01) | 56* | 0.87 (0.80, 0.94)** | 15 | 1.03 (0.98, 1.09) | 20 | 1.07 (1.02, 1.11)** | 11 | 0.97 (0.87, 1.08) | 34 | 0.98 (0.89, 1.06) | 25 |
| Np/Nc | 21063/7 |  | 20877/7 |  | 21434/7 |  | 21298/7 |  | 21425/7 |  | 21471/7 |  |
| Early | 0.79 (0.65, 0.97)* | 45 | 0.81 (0.73, 0.89)*** | 0 | 1.04 (0.97, 1.12) | 16 | 1.06 (0.99, 1.14) | 30 | 0.96 (0.83, 1.10) | 27 | 0.95 (0.82, 1.09) | 28 |
| Np/Nc | 9938/5 |  | 9793/5 |  | 10359/5 |  | 10214/5 |  | 10176/5 |  | 10222/5 |  |
| Late | 0.96 (0.85, 1.09) | 12 | 0.88 (0.77, 1.002) | 40 | 1.04 (0.96, 1.14) | 34 | 1.07 (1.02, 1.12)** | 0 | 0.93 (0.77, 1.12) | 61 | 0.95 (0.82, 1.10) | 44 |
| Np/Nc | 13447/3 |  | 13406/3 |  | 13397/3 |  | 13406/3 |  | 13587/3 |  | 13587/3 |  |

Values are adjusted pooled effect estimates [OR (95% CI)] expressed for a 1-SD increment in dietary scores, heterogeneity measure (*I*^2^), and number of participants and studies included (Np/Nc) across different outcomes and conception periods, as labelled. Effect estimates were adjusted for maternal education, pre-pregnancy BMI, ethnicity, maternal height, parity, energy intake (for DASH), cigarette smoking and alcohol consumption during pregnancy, and child sex.

E-DII, energy-adjusted Dietary Inflammatory Index; DASH, Dietary Approaches to Stop Hypertension; *I*^2^, *I*-squared; SGA, small-for-gestational-age; LGA, large-for-gestational-age; Pre, pre-pregnancy; Preg, pregnancy; Early, early pregnancy; Late, late pregnancy; Np, number of participants included; Nc, number of cohorts included.

**P*<0.05, ***P*<0.01, ****P*<0.001
