# Supplementary material for: Associations of maternal dietary inflammatory potential and quality with offspring birth outcomes: An individual participant data pooled analysis of 7 European cohorts in the ALPHABET consortium
Source: PLoS Med. 2021 Jan 21;18(1):e1003491. doi: 10.1371/journal.pmed.1003491 (PMC7819611; doi:10.1371/journal.pmed.1003491)
Supplement: S11 Table — (DOCX) [file pmed.1003491.s013.docx]

**S11 Table** Sensitivity analysis for gestational age outcomes- restricting samples to all spontaneous labours

|  | Gestational age, wk |  |  | Preterm birth |  | Post-term birth |  |
| --- | --- | --- | --- | --- | --- | --- | --- |
|  | β (95%CI) | *I^2^ (%)* |  | OR (95% CI) | *I^2^ (%)* | OR (95% CI) | *I^2^ (%)* |
| **E-DII** |  |  |  |  |  |  |  |
| *Pre* | -0.01 (-0.07, 0.06) | 0 |  | 0.93 (0.77, 1.13) | 21 | 1.05 (0.64, 1.70) | 52 |
| Np/Nc | 3047/2 |  |  | 3015/2 |  | 3047/2 |  |
| *Preg* | -0.02 (-0.07, 0.03) | 70** |  | 0.997 (0.87, 1.14) | 55* | 0.97 (0.91, 1.04) | 5 |
| Np/Nc | 21099/7 |  |  | 20961/7 |  | 21027/7 |  |
| *Early* | -0.01 (-0.09, 0.07) | 73** |  | 1.10 (0.995, 1.21) | 0 | 1.02 (0.90, 1.15) | 7 |
| Np/Nc | 9578/5 |  |  | 9473/5 |  | 9506/5 |  |
| *Late* | -0.01 (-0.07, 0.05) | 61 |  | 0.94 (0.73, 1.22) | 76* | 1.03 (0.83, 1.29) | 40 |
| Np/Nc | 13385/3 |  |  | 13352/3 |  | 13385/3 |  |
|  |  |  |  |  |  |  |  |
| **DASH** |  |  |  |  |  |  |  |
| *Pre* | -0.01 (-0.09, 0.07) | 32 |  | 1.03 (0.84, 1.26) | 22 | 0.82 (0.52, 1.31) | 41 |
| Np/Nc | 3047/2 |  |  | 3015/2 |  | 3047/2 |  |
| *Preg* | 0.02 (-0.02, 0.05) | 45 |  | 0.97 (0.89, 1.06) | 17 | 0.98 (0.88, 1.10) | 33 |
| Np/Nc | 21098/7 |  |  | 20960/7 |  | 21026/7 |  |
| *Early* | 0.02 (-0.04, 0.07) | 48 |  | 1.01 (0.87, 1.17) | 28 | 0.95 (0.82, 1.12) | 16 |
| Np/Nc | 9577/5 |  |  | 9472/5 |  | 9505/5 |  |
| *Late* | 0.01 (-0.02, 0.05) | 17 |  | 0.96 (0.88, 1.06) | 0 | 0.89 (0.66, 1.19) | 58 |
| Np/Nc | 13384/3 |  |  | 13351/3 |  | 13384/3 |  |

Values are adjusted pooled effect estimates [β or OR (95% CI)] expressed for a 1-SD increment in dietary scores, heterogeneity measure (*I*^2^), and number of participants and studies included (Np/Nc) across different outcomes and conception periods, as labelled. Effect estimates were adjusted for maternal education, pre-pregnancy BMI, ethnicity, maternal height, parity, energy intake (for DASH), cigarette smoking and alcohol consumption during pregnancy, and child sex.

E-DII, energy-adjusted Dietary Inflammatory Index; DASH, Dietary Approaches to Stop Hypertension; *I*^2^, *I*-squared; Pre, pre-pregnancy; Preg, pregnancy; Early, early pregnancy; Late, late pregnancy; Np, number of participants included; Nc, number of cohorts included.

**P*<0.05, ***P*<0.01, ****P*<0.001
