# Supplementary material for: Associations of maternal dietary inflammatory potential and quality with offspring birth outcomes: An individual participant data pooled analysis of 7 European cohorts in the ALPHABET consortium
Source: PLoS Med. 2021 Jan 21;18(1):e1003491. doi: 10.1371/journal.pmed.1003491 (PMC7819611; doi:10.1371/journal.pmed.1003491)
Supplement: S12 Table — (DOCX) [file pmed.1003491.s014.docx]

**S12 Table** Sensitivity analysis for primary birth size measure- restricting samples born between 37 to <42 completed weeks of gestational age

|  | Birthweight, g |  |  | Low birth weight |  | SGA |  | Macrosomia |  | LGA |  |
| --- | --- | --- | --- | --- | --- | --- | --- | --- | --- | --- | --- |
|  | β (95%CI) | *I^2^ (%)* |  | OR (95% CI) | *I^2^ (%)* | OR (95% CI) | *I^2^ (%)* | OR (95% CI) | *I^2^ (%)* | OR (95% CI) | *I^2^ (%)* |
| **E-DII** |  |  |  |  |  |  |  |  |  |  |  |
| Pre | -16.5 (-30.8, -2.2)* | 0 |  | 1.21 (0.86, 1.69) | 39 | 1.15 (0.98, 1.34) | 0 | 0.97 (0.87, 1.09) | 0 | 0.96 (0.88, 1.05) | 0 |
| Np/Nc | 3750/2 |  |  | 3750/2 |  | 3750/2 |  | 3699/2 |  | 3750/2 |  |
| Preg | -17.0 (-29.1, -4.9)** | 61* |  | 1.33 (1.15, 1.53)*** | 30 | 1.21 (1.13, 1.29)*** | 0 | 0.95 (0.88, 1.02) | 51 | 0.97 (0.91, 1.05) | 61* |
| Np/Nc | 21367/7 |  |  | 20833/6 |  | 20720/6 |  | 21315/7 |  | 21253/7 |  |
| Early | -11.5 (-31.7, 8.6) | 71** |  | 1.38 (1.09, 1.74)** | 41 | 1.18 (1.07, 1.31)** | 0 | 0.99 (0.90, 1.09) | 48 | 1.01 (0.91, 1.12) | 66* |
| Np/Nc | 9729/5 |  |  | 9195/4 |  | 9082/4 |  | 9729/5 |  | 9615/5 |  |
| Late | -19.3 (-30.2, -8.4)** | 33 |  | 1.17 (0.89, 1.52) | 57 | 1.21 (1.11, 1.32)*** | 0 | 0.92 (0.82, 1.02) | 55 | 0.95 (0.87, 1.04) | 57* |
| Np/Nc | 13885/3 |  |  | 13885/3 |  | 13885/3 |  | 13833/3 |  | 13885/3 |  |
|  |  |  |  |  |  |  |  |  |  |  |  |
| **DASH** |  |  |  |  |  |  |  |  |  |  |  |
| Pre | 16.4 (1.7, 31.2)* | 0 |  | 0.89 (0.64, 1.24) | 35 | 0.86 (0.73, 1.003) | 0 | 1.05 (0.93, 1.17) | 0 | 1.06 (0.97, 1.16) | 0 |
| Np/Nc | 3750/2 |  |  | 3750/2 |  | 3750/2 |  | 3699/2 |  | 3750/2 |  |
| Preg | 18.0 (5.5, 30.5)** | 62* |  | 0.79 (0.66, 0.94)** | 48 | 0.87 (0.78, 0.97)* | 45 | 1.03 (0.96, 1.12) | 54* | 1.06 (0.99, 1.13) | 57* |
| Np/Nc | 21365/7 |  |  | 20832/6 |  | 20719/6 |  | 21313/7 |  | 21252/7 |  |
| Early | 19.6 (3.9, 35.4)* | 50 |  | 0.73 (0.55, 0.98)* | 55 | 0.82 (0.73, 0.91)*** | 0 | 1.03 (0.95, 1.12) | 30 | 1.05 (0.97, 1.13) | 42 |
| Np/Nc | 9727/5 |  |  | 9194/4 |  | 9081/4 |  | 9727/5 |  | 9614/5 |  |
| Late | 16.9 (3.1, 30.7)* | 51 |  | 0.88 (0.77, 1.01) | 0 | 0.86 (0.72, 1.03) | 66 | 1.09 (0.94, 1.27) | 73* | 1.10 (0.97, 1.24) | 73* |
| Np/Nc | 13884/3 |  |  | 13884/3 |  | 13884/3 |  | 13832/3 |  | 13884/3 |  |

Values are adjusted pooled effect estimates [β or OR (95% CI)] expressed for a 1-SD increment in dietary scores, heterogeneity measure (*I*^2^), and number of participants and studies included (Np/Nc) across different outcomes and conception periods, as labelled. Effect estimates were adjusted for maternal education, pre-pregnancy BMI, ethnicity, maternal height, parity, energy intake (for DASH), cigarette smoking and alcohol consumption during pregnancy, and child sex.

E-DII, energy-adjusted Dietary Inflammatory Index; DASH, Dietary Approaches to Stop Hypertension; *I*^2^, *I*-squared; SGA, small-for-gestational-age; LGA, large-for-gestational-age; Pre, pre-pregnancy; Preg, pregnancy; Early, early pregnancy; Late, late pregnancy; Np, number of participants included; Nc, number of cohorts included.

**P*<0.05, ***P*<0.01, ****P*<0.001
