# Supplementary material for: Associations of maternal dietary inflammatory potential and quality with offspring birth outcomes: An individual participant data pooled analysis of 7 European cohorts in the ALPHABET consortium
Source: PLoS Med. 2021 Jan 21;18(1):e1003491. doi: 10.1371/journal.pmed.1003491 (PMC7819611; doi:10.1371/journal.pmed.1003491)
Supplement: S13 Table — (DOCX) [file pmed.1003491.s015.docx]

**S13 Table** Sensitivity analysis for primary birth size measure- restricting samples born between 39 to <41 completed weeks of gestational age

|  | Birthweight, g |  |  | Low birth weight |  | SGA |  | Macrosomia |  | LGA |  |
| --- | --- | --- | --- | --- | --- | --- | --- | --- | --- | --- | --- |
|  | β (95%CI) | *I^2^ (%)* |  | OR (95% CI) | *I^2^ (%)* | OR (95% CI) | *I^2^ (%)* | OR (95% CI) | *I^2^ (%)* | OR (95% CI) | *I^2^ (%)* |
| **E-DII** |  |  |  |  |  |  |  |  |  |  |  |
| Pre | -12.2 (-29.7, 5.2) | 0 |  | 1.49 (0.80, 2.76) | 0 | 1.17 (0.94, 1.44) | 0 | 1.01 (0.87, 1.19) | 0 | 0.98 (0.87, 1.10) | 0 |
| Np/Nc | 2183/2 |  |  | 2153/2 |  | 2153/2 |  | 2153/2 |  | 2183/2 |  |
| Preg | -19.5 (-30.0, -9.0)*** | 30 |  | 1.57 (1.28, 1.93)*** | 0 | 1.26 (1.15, 1.38)*** | 0 | 0.94 (0.87, 1.02) | 30 | 0.94 (0.89, 1.003) | 24 |
| Np/Nc | 12502/7 |  |  | 12146/6 |  | 12033/6 |  | 12471/7 |  | 12388/7 |  |
| Early | -13.9 (-30.0, 2.2) | 36 |  | 1.50 (1.12, 1.99)** | 0 | 1.22 (1.06, 1.40) | 0 | 0.97 (0.87, 1.08) | 32 | 0.96 (0.89, 1.04) | 14 |
| Np/Nc | 5855/5 |  |  | 5530/4 |  | 5417/4 |  | 5855/5 |  | 5741/5 |  |
| Late | -21.1 (-34.5, -7.6)** | 33 |  | 1.61 (1.21, 2.13)** | 0 | 1.29 (1.15, 1.45)*** | 0 | 0.93 (0.84, 1.02) | 20 | 0.95 (0.85, 1.05) | 50 |
| Np/Nc | 7928/3 |  |  | 7897/3 |  | 7897/3 |  | 7897/3 |  | 7928/3 |  |
|  |  |  |  |  |  |  |  |  |  |  |  |
| **DASH** |  |  |  |  |  |  |  |  |  |  |  |
| Pre | 16.1 (-1.9, 34.2) | 0 |  | 1.01 (0.55, 1.87) | 0 | 0.84 (0.68, 1.04) | 0 | 0.98 (0.83, 1.15) | 0 | 1.06 (0.94, 1.20) | 0 |
| Np/Nc | 2183/2 |  |  | 2153/2 |  | 2153/2 |  | 2153/2 |  | 2183/2 |  |
| Preg | 13.2 (5.1, 21.3)** | 4 |  | 0.68 (0.56, 0.84)*** | 0 | 0.85 (0.74, 0.98)* | 39 | 1.004 (0.95, 1.07) | 0 | 1.04 (0.99, 1.09) | 0 |
| Np/Nc | 12500/7 |  |  | 12145/6 |  | 12032/6 |  | 12469/7 |  | 12387/7 |  |
| Early | 11.3 (-3.4, 25.9) | 23 |  | 0.62 (0.43, 0.89)* | 20 | 0.79 (0.69, 0.92)** | 0 | 0.99 (0.91, 1.07) | 0 | 1.02 (0.95, 1.09) | 0 |
| Np/Nc | 5853/5 |  |  | 5529/4 |  | 5416/4 |  | 5853/5 |  | 5740/5 |  |
| Late | 12.4 (2.9, 21.9)* | 0 |  | 0.78 (0.59, 1.01) | 0 | 0.80 (0.61, 1.06) | 73* | 1.07 (0.93, 1.23) | 45 | 1.06 (0.994, 1.12) | 0 |
| Np/Nc | 7928/3 |  |  | 7897/3 |  | 7897/3 |  | 7897/3 |  | 7928/3 |  |

Values are adjusted pooled effect estimates [β or OR (95% CI)] expressed for a 1-SD increment in dietary scores, heterogeneity measure (*I*^2^), and number of participants and studies included (Np/Nc) across different outcomes and conception periods, as labelled. Effect estimates were adjusted for maternal education, pre-pregnancy BMI, ethnicity, maternal height, parity, energy intake (for DASH), cigarette smoking and alcohol consumption during pregnancy, and child sex.

E-DII, energy-adjusted Dietary Inflammatory Index; DASH, Dietary Approaches to Stop Hypertension; *I*^2^, *I*-squared; SGA, small-for-gestational-age; LGA, large-for-gestational-age; Pre, pre-pregnancy; Preg, pregnancy; Early, early pregnancy; Late, late pregnancy; Np, number of participants included; Nc, number of cohorts included.

**P*<0.05, ***P*<0.01, ****P*<0.001
