# Supplementary material for: Associations of maternal dietary inflammatory potential and quality with offspring birth outcomes: An individual participant data pooled analysis of 7 European cohorts in the ALPHABET consortium
Source: PLoS Med. 2021 Jan 21;18(1):e1003491. doi: 10.1371/journal.pmed.1003491 (PMC7819611; doi:10.1371/journal.pmed.1003491)
Supplement: S14 Table — (DOCX) [file pmed.1003491.s016.docx]

**S14 Table** Sensitivity analysis for continuous birth size measure not intrinsically adjusted for gestational age- further adjusting for gestational age to assess potential mediation

|  | Birthweight, g |  | Birth length, cm |  | Head circumference, cm |  |
| --- | --- | --- | --- | --- | --- | --- |
|  | β (95%CI) | *I^2^ (%)* | β (95%CI) | *I^2^ (%)* | β (95%CI) | *I^2^ (%)* |
| **E-DII** |  |  |  |  |  |  |
| Pre | -15.1 (-29.3, -0.9)* | 20 | -0.07 (-0.12, -0.01)* | 0 | -0.03 (-0.07, 0.01) | 0 |
| Np/Nc | 4119/2 |  | 3964/2 |  | 3993/2 |  |
| Preg | -13.4 (-23.6, -3.1)* | 57* | -0.04 (-0.09, 0.01) | 52 | -0.03 (-0.07, 0.01) | 61* |
| Np/Nc | 23879/7 |  | 18861/7 |  | 18242/7 |  |
| Early | -7.9 (-25.5, 9.7) | 68* | -0.02 (-0.11, 0.07) | 66* | -0.01 (-0.08, 0.06) | 72** |
| Np/Nc | 10749/5 |  | 8289/5 |  | 7549/5 |  |
| Late | -17.5 (-24.4, -10.6)*** | 0 | -0.04 (-0.08, -0.01)* | 0 | -0.02 (-0.05, -0.001)* | 0 |
| Np/Nc | 15621/3 |  | 12955/3 |  | 13104/3 |  |
|  |  |  |  |  |  |  |
| **DASH** |  |  |  |  |  |  |
| Pre | 19.1 (6.0, 32.1)** | 0 | 0.08 (0.02, 0.13)** | 0 | 0.01 (-0.03, 0.05) | 0 |
| Np/Nc | 4119/2 |  | 3964/2 |  | 3993/2 |  |
| Preg | 15.2 (5.6, 24.8)** | 49 | 0.04 (-0.001, 0.09) | 37 | 0.02 (0.003, 0.05)* | 0 |
| Np/Nc | 23878/7 |  | 18860/7 |  | 18242/7 |  |
| Early | 18.1 (7.5, 28.6)** | 20 | 0.07 (0.02, 0.11)** | 0 | 0.03 (-0.00, 0.07) | 0 |
| Np/Nc | 10748/5 |  | 8288/5 |  | 7549/5 |  |
| Late | 15.9 (2.3, 29.5)* | 59 | 0.05 (-0.03, 0.12) | 71* | 0.02 (-0.003, 0.04) | 0 |
| Np/Nc | 15620/3 |  | 12954/3 |  | 13103/3 |  |

Values are adjusted pooled effect estimates [β (95% CI)] expressed for a 1-SD increment in dietary scores, heterogeneity measure (*I*^2^), and number of participants and studies included (Np/Nc) across different outcomes and conception periods, as labelled. Effect estimates were adjusted for maternal education, pre-pregnancy BMI, ethnicity, maternal height, parity, energy intake (for DASH), cigarette smoking and alcohol consumption during pregnancy, child sex, and gestational age.

E-DII, energy-adjusted Dietary Inflammatory Index; DASH, Dietary Approaches to Stop Hypertension; *I*^2^, *I*-squared; SGA, small-for-gestational-age; LGA, large-for-gestational-age; Pre, pre-pregnancy; Preg, pregnancy; Early, early pregnancy; Late, late pregnancy; Np, number of participants included; Nc, number of cohorts included.

**P*<0.05, ***P*<0.01, ****P*<0.001
