# Supplementary material for: Associations of maternal dietary inflammatory potential and quality with offspring birth outcomes: An individual participant data pooled analysis of 7 European cohorts in the ALPHABET consortium
Source: PLoS Med. 2021 Jan 21;18(1):e1003491. doi: 10.1371/journal.pmed.1003491 (PMC7819611; doi:10.1371/journal.pmed.1003491)
Supplement: S15 Table — (DOCX) [file pmed.1003491.s017.docx]

**S15 Table** Comparison of results with different imputation methods for covariates

|  | Continuous |  |  |  | Binary |  |  |  |  |  |
| --- | --- | --- | --- | --- | --- | --- | --- | --- | --- | --- |
|  | BW | GA | BL | HC | LBW | SGA | Macrosomia | LGA | Preterm | Post-term |
| **Pre-pregnancy** |  |  |  |  |  |  |  |  |  |  |
| E-DII |  |  |  |  |  |  |  |  |  |  |
| Single imputation | -18.7 (-34.8, -2.6) | -0.02 (-0.08, 0.04) | -0.07 (-0.14, -0.01) | -0.04 (-0.10, 0.03) | 1.16 (0.99, 1.36) | 1.14 (0.99, 1.32) | 0.99 (0.89, 1.10) | 0.97 (0.89, 1.05) | 1.04 (0.90, 1.19) | 1.10 (0.92, 1.31) |
| Multiple imputation | -15.9 (-32.2, 0.3) | -0.01 (-0.07, 0.05) | -0.07 (-0.14, -0.001) | -0.03 (-0.10, 0.03) | 1.15 (0.97, 1.32) | 1.13 (0.97, 1.30) | 0.998 (0.89, 1.11) | 0.98 (0.83, 1.13) | 1.01 (0.87, 1.15) | 1.09 (0.90, 1.28) |
| DASH |  |  |  |  |  |  |  |  |  |  |
| Single imputation | 18.6 (-3.0, 40.3) | 0.001 (-0.06, 0.06) | 0.07 (0.004, 0.14) | 0.01 (-0.04, 0.06) | 0.88 (0.61, 1.28) | 0.85 (0.71, 1.02) | 1.05 (0.94, 1.17) | 1.05 (0.92, 1.20) | 0.95 (0.82, 1.10) | 0.90 (0.67, 1.20) |
| Multiple imputation | 23.8 (6.5, 41.1) | 0.01 (-0.07, 0.09) | 0.07 (-0.01, 0.15) | 0.02 (-0.03, 0.07) | 0.91 (0.74, 1.09) | 0.85 (0.71, 0.98) | 1.07 (0.93, 1.20) | 1.17 (1.06, 1.29) | 0.88 (0.73, 1.04) | 0.84 (0.56, 1.13) |
|  |  |  |  |  |  |  |  |  |  |  |
| **Pregnancy** |  |  |  |  |  |  |  |  |  |  |
| E-DII |  |  |  |  |  |  |  |  |  |  |
| Single imputation | -14.3 (-29.3, 0.7) | -0.02 (-0.07, 0.03) | -0.06 (-0.10, -0.01) | -0.03 (-0.07, 0.01) | 1.14 (1.04, 1.26) | 1.18 (1.11, 1.26) | 0.95 (0.89, 1.02) | 0.98 (0.92, 1.05) | 1.02 (0.92, 1.13) | 0.99 (0.92, 1.06) |
| Multiple imputation | -13.8 (-28.8, 1.3) | -0.02 (-0.06, 0.03) | -0.05 (-0.10, -0.001) | -0.03 (-0.07, 0.01) | 1.12 (0.996, 1.24) | 1.18 (1.10, 1.25) | 0.94 (0.88, 1.01) | 0.97 (0.91, 1.03) | 1.004 (0.92, 1.02) | 0.97 (0.92, 1.02) |
| DASH |  |  |  |  |  |  |  |  |  |  |
| Single imputation | 18.5 (5.7, 31.3) | 0.02 (-0.01, 0.05) | 0.05 (0.01, 0.10) | 0.03 (0.01, 0.06) | 0.89 (0.82, 0.95) | 0.87 (0.82, 0.94) | 1.03 (0.97, 1.10) | 1.05 (0.99, 1.12) | 0.96 (0.89, 1.04) | 0.98 (0.89, 1.08) |
| Multiple imputation | 17.6 (4.8, 30.5) | 0.02 (-0.01, 0.05) | 0.05 (0.01, 0.09) | 0.03 (0.01, 0.05) | 0.88 (0.81, 0.94) | 0.87 (0.81, 0.93) | 1.02 (0.96, 1.08) | 1.05 (0.99, 1.11) | 0.95 (0.89, 1.02) | 0.95 (0.85, 1.05) |

Values are adjusted pooled effect estimates [β (95% CI)] for continuous outcomes or [OR (95% CI)] for binary outcomes, expressed for a 1-SD increment in dietary scores across different outcomes and conception periods, as labelled. Effect estimates were adjusted for maternal education, pre-pregnancy BMI, maternal height, parity, energy intake (for DASH), cigarette smoking and alcohol consumption during pregnancy, and child sex.

E-DII, energy-adjusted Dietary Inflammatory Index; DASH, Dietary Approaches to Stop Hypertension; BW, birth weight; GA, gestational age; BL, birth length; HC, head circumference; LBW, low birth weight; SGA, small for gestational age; LGA, large for gestational age
