# Supplementary material for: Associations of maternal dietary inflammatory potential and quality with offspring birth outcomes: An individual participant data pooled analysis of 7 European cohorts in the ALPHABET consortium
Source: PLoS Med. 2021 Jan 21;18(1):e1003491. doi: 10.1371/journal.pmed.1003491 (PMC7819611; doi:10.1371/journal.pmed.1003491)
Supplement: S16 Table — (DOCX) [file pmed.1003491.s018.docx]

**S16 Table** Sensitivity analysis for continuous outcomes- mutually adjusting for dietary scores

|  | Primary outcomes | | | |  | Secondary outcomes | | | | | | | |
| --- | --- | --- | --- | --- | --- | --- | --- | --- | --- | --- | --- | --- | --- |
|  | Birthweight, g |  | Gestational age, wk |  |  | Birth length, cm |  | Head circumference, cm |  | Abdominal circumference, cm |  | Sum of skinfold thickness, mm |  |
|  | β (95%CI) | *I^2^ (%)* | β (95%CI) | *I^2^ (%)* |  | β (95%CI) | *I^2^ (%)* | β (95%CI) | *I^2^ (%)* | β (95%CI) | *I^2^ (%)* | β (95%CI) | *I^2^ (%)* |
| **E-DII** |  |  |  |  |  |  |  |  |  |  |  |  |  |
| *Pre* | -9.9 (-30.4, 10.5) | 0 | -0.02 (-0.13, 0.09) | 57 |  | -0.04 (-0.13, 0.04) | 0 | -0.03 (-0.12, 0.06) | 49 | 0.11 (0.01, 0.22)* | - | 0.01 (-0.06, 0.09) | 0 |
| Np/Nc | 4119/2 |  | 4137/2 |  |  | 3964/2 |  | 3993/2 |  | 2406/1 |  | 3923/2 |  |
| *Preg* | -8.5 (-28.6, 11.7) | 77*** | -0.02 (-0.08, 0.04) | 73** |  | -0.03 (-0.12, 0.06) | 65** | -0.02 (-0.07, 0.02) | 50 | 0.11 (-0.01, 0.23) | 0 | 0.02 (-0.07, 0.11) | 17 |
| Np/Nc | 23991/7 |  | 24100/7 |  |  | 18952/7 |  | 18333/7 |  | 2030/2 |  | 3530/3 |  |
| *Early* | -8.3 (-36.7, 20.1) | 74** | -0.05 (-0.12, 0.03) | 65* |  | -0.03 (-0.15, 0.09) | 61* | -0.02 (-0.09, 0.04) | 45 | 0.06 (-0.06, 0.17) | 0 | -0.08 (-0.38, 0.21) | 44 |
| Np/Nc | 10861/5 |  | 10824/5 |  |  | 8380/5 |  | 7640/5 |  | 2128/2 |  | 2081/2 |  |
| *Late* | -3.1 (-35.8, 29.6) | 85** | 0.03 (-006, 0.13) | 81** |  | -0.01 (-0.12, 0.11) | 76* | -0.02 (-0.08, 0.05) | 64 | 0.08 (-0.03, 0.19) | - | 0.05 (-0.03, 0.12) | 0 |
| Np/Nc | 15620/3 |  | 15783/3 |  |  | 12954/3 |  | 13103/3 |  | 2308/1 |  | 3841/2 |  |
|  |  |  |  |  |  |  |  |  |  |  |  |  |  |
| **DASH** |  |  |  |  |  |  |  |  |  |  |  |  |  |
| *Pre* | 12.5 (-16.1, 41.0) | 47 | -0.01 (-0.13, 0.11) | 63 |  | 0.05 (-0.04, 0.13) | 0 | -0.004 (-0.06, 0.05) | 0 | 0.16 (0.05, 0.27) | - | 0.07 (0.002, 0.14)* | 0 |
| Np/Nc | 4119/2 |  | 4137/2 |  |  | 3964/2 |  | 3993/2 |  | 2406/1 |  | 3923/2 |  |
| *Preg* | 13.1 (-1.8, 28.0) | 57* | 0.01 (-0.02, 0.03) | 0 |  | 0.04 (-0.02, 0.11) | 47 | 0.02 (-0.01, 0.05) | 0 | 0.09 (-0.14, 0.31) | 42 | 0.07 (-0.03, 0.17) | 28 |
| Np/Nc | 23991/7 |  | 24100/7 |  |  | 18952/7 |  | 18333/7 |  | 2030/2 |  | 3530/3 |  |
| *Early* | 14.9 (-1.5, 31.3) | 28 | -0.001 (-0.04, 0.04) | 0 |  | 0.05 (-0.01, 0.12) | 0 | 0.02 (-0.02, 0.07) | 0 | 0.08 (-0.06, 0.21) | 9 | 0.11 (0.01, 0.21)* | 0 |
| Np/Nc | 10861/5 |  | 10824/5 |  |  | 8380/5 |  | 7640/5 |  | 2128/2 |  | 2081/2 |  |
| *Late* | 18.8 (-10.2, 47.9) | 81** | 0.03 (-0.02, 0.09) | 45 |  | 0.06 (-0.07, 0.19) | 80** | 0.02 (-0.04, 0.08) | 64 | 0.15 (0.05, 0.26)** | - | 0.07 (-0.02, 0.15) | 25 |
| Np/Nc | 15620/3 |  | 15783/3 |  |  | 12954/3 |  | 13103/3 |  | 2308/1 |  | 3841/2 |  |

Values are adjusted pooled effect estimates [β (95% CI)] expressed for a 1-SD increment in dietary scores, heterogeneity measure (*I*^2^), and number of participants and studies included (Np/Nc) across different outcomes and conception periods, as labelled. Effect estimates were adjusted for maternal education, pre-pregnancy BMI, maternal height, parity, energy intake (for DASH), cigarette smoking and alcohol consumption during pregnancy, and child sex. Dietary scores were mutually adjusted in this model.

E-DII, energy-adjusted Dietary Inflammatory Index; DASH, Dietary Approaches to Stop Hypertension; *I*^2^, *I*-squared; Pre, pre-pregnancy; Preg, pregnancy; Early, early pregnancy; Late, late pregnancy; Np, number of participants included; Nc, number of cohorts included.

**P*<0.05, ***P*<0.01, ****P*<0.001
