# Supplementary material for: Associations of maternal dietary inflammatory potential and quality with offspring birth outcomes: An individual participant data pooled analysis of 7 European cohorts in the ALPHABET consortium
Source: PLoS Med. 2021 Jan 21;18(1):e1003491. doi: 10.1371/journal.pmed.1003491 (PMC7819611; doi:10.1371/journal.pmed.1003491)
Supplement: S17 Table — (DOCX) [file pmed.1003491.s019.docx]

**S17 Table** Sensitivity analysis for binary outcomes- mutually adjusting for dietary scores

|  | Low birth weight |  | SGA |  | Macrosomia |  | LGA |  | Preterm birth |  | Post-term birth |  |
| --- | --- | --- | --- | --- | --- | --- | --- | --- | --- | --- | --- | --- |
|  | OR (95% CI) | *I^2^ (%)* | OR (95% CI) | *I^2^ (%)* | OR (95% CI) | *I^2^ (%)* | OR (95% CI) | *I^2^ (%)* | OR (95% CI) | *I^2^ (%)* | OR (95% CI) | *I^2^ (%)* |
| **E-DII** |  |  |  |  |  |  |  |  |  |  |  |  |
| Pre | 1.13 (0.92, 1.37) | 0 | 1.07 (0.89, 1.29) | 0 | 1.02 (0.89, 1.17) | 0 | 1.002 (0.90, 1.17) | 0 | 1.01 (0.85, 1.21) | 0 | 1.06 (0.85, 1.33) | 0 |
| Np/Nc | 4119/2 |  | 4119/2 |  | 4065/2 |  | 4119/2 |  | 4137/2 |  | 4137/2 |  |
| Preg | 1.09 (0.91, 1.31) | 67* | 1.16 (1.05, 1.27)** | 21 | 0.95 (0.87, 1.04) | 55* | 1.01 (0.92, 1.10) | 67** | 1.003 (0.84, 1.12) | 68** | 0.96 (0.90, 1.03) | 0 |
| Np/Nc | 23419/6 |  | 23218/6 |  | 23936/7 |  | 23790/7 |  | 23952/7 |  | 23997/7 |  |
| Early | 1.19 (0.997, 1.42) | 31 | 1.12 (0.98, 1.27) | 5 | 0.99 (0.86, 1.13) | 63* | 1.03 (0.91, 1.18) | 69* | 1.14 (1.02, 1.28)* | 0 | 0.96 (0.86, 1.07) | 0 |
| Np/Nc | 10289/4 |  | 10139/4 |  | 10861/5 |  | 10711/5 |  | 10676/5 |  | 10721/5 |  |
| Late | 0.97 (0.70, 1.34) | 81** | 1.10 (0.89, 1.36) | 67* | 0.95 (0.81, 1.12) | 70* | 0.999 (0.88, 1.14) | 72* | 0.88 (0.66, 1.17) | 80** | 0.97 (0.90, 1.05) | 0 |
| Np/Nc | 15620/3 |  | 15569/3 |  | 15565/3 |  | 15569/3 |  | 15783/3 |  | 15783/3 |  |
|  |  |  |  |  |  |  |  |  |  |  |  |  |
| **DASH** |  |  |  |  |  |  |  |  |  |  |  |  |
| Pre | 0.95 (0.59, 1.54) | 82* | 0.89 (0.70, 1.12) | 39 | 1.06 (0.92, 1.21) | 0 | 1.05 (0.87, 1.26) | 61 | 0.96 (0.80, 1.14) | 0 | 0.96 (0.74, 1.24) | 18 |
| Np/Nc | 4119/2 |  | 4119/2 |  | 4065/2 |  | 4119/2 |  | 4137/2 |  | 4137/2 |  |
| Preg | 0.92 (0.80, 1.06) | 48 | 0.94 (0.82, 1.07) | 51 | 1.003 (0.93, 1.08) | 41 | 1.06 (0.98, 1.14) | 52 | 0.97 (0.84, 1.13) | 55* | 0.99 (0.91, 1.07) | 9 |
| Np/Nc | 23419/6 |  | 23218/6 |  | 23936/7 |  | 23790/7 |  | 23952/7 |  | 23997/7 |  |
| Early | 0.90 (0.71, 1.15) | 59 | 0.92 (0.76, 1.10) | 39 | 1.02 (0.92, 1.12) | 32 | 1.06 (0.98, 1.14) | 17 | 1.06 (0.87, 1.28) | 42 | 0.94 (0.85, 1.05) | 0 |
| Np/Nc | 10289/5 |  | 10139/4 |  | 10861/5 |  | 10711/5 |  | 10676/5 |  | 10721/5 |  |
| Late | 0.91 (0.78, 1.08) | 33 | 0.89 (0.71, 1.13) | 74* | 1.05 (0.90, 1.23) | 68* | 1.11 (0.94, 1.29) | 79** | 0.87 (0.71, 1.05) | 57 | 1.01 (0.94, 1.09) | 0 |
| Np/Nc | 15620/3 |  | 15569/3 |  | 15565/3 |  | 15569/3 |  | 15783/3 |  | 15783/3 |  |

Values are adjusted pooled effect estimates [OR (95% CI)] expressed for a 1-SD increment in dietary scores, heterogeneity measure (*I*^2^), and number of participants and studies included (Np/Nc) across different outcomes and conception periods, as labelled. Effect estimates were adjusted for maternal education, pre-pregnancy BMI, maternal height, parity, energy intake (for DASH), cigarette smoking and alcohol consumption during pregnancy, and child sex. Dietary scores were mutually adjusted in this model.

E-DII, energy-adjusted Dietary Inflammatory Index; DASH, Dietary Approaches to Stop Hypertension; *I*^2^, *I*-squared; SGA, small-for-gestational-age; LGA, large-for-gestational-age; Pre, pre-pregnancy; Preg, pregnancy; Early, early pregnancy; Late, late pregnancy; Np, number of participants included; Nc, number of cohorts included.

**P*<0.05, ***P*<0.01, ****P*<0.001
