# Supplementary material for: Associations of maternal dietary inflammatory potential and quality with offspring birth outcomes: An individual participant data pooled analysis of 7 European cohorts in the ALPHABET consortium
Source: PLoS Med. 2021 Jan 21;18(1):e1003491. doi: 10.1371/journal.pmed.1003491 (PMC7819611; doi:10.1371/journal.pmed.1003491)
Supplement: S18 Table — (DOCX) [file pmed.1003491.s020.docx]

**S18 Table** Quartile estimates for non-linear relationships

|  | Q1 | Q2 | Q3 | Q4 |
| --- | --- | --- | --- | --- |
| **OR (95% CI)** |  |  |  |  |
| Early pregnancy DASH vs. LBW | Reference | 0.67 (0.52, 0.87)** | 0.57 (0.41, 0.79)** | 0.72 (0.50, 1.03) |
|  |  |  |  |  |
| **Β (95% CI)** |  |  |  |  |
| Pre-pregnancy DASH vs. birth length | Reference | 0.10 (-0.08, 0.28) | 0.08 (-0.11, 0.26) | 0.15 (-0.04, 0.34) |
| Pre-pregnancy DASH vs. abdominal circumference | Reference | 0.23 (0.00, 0.45) | 0.19 (-0.04, 0.43) | 0.22 (-0.03, 0.46) |

Values are adjusted pooled effect estimates [β (95% CI)] or [OR (95% CI)] across different outcomes as labelled. Effect estimates were adjusted for maternal education, ethnicity, pre-pregnancy BMI, maternal height, parity, energy intake (for DASH), cigarette smoking and alcohol consumption during pregnancy, and child sex.

E-DII, energy-adjusted Dietary Inflammatory Index; DASH, Dietary Approaches to Stop Hypertension; LBW, low birth weight
