# Supplementary material for: Associations of maternal dietary inflammatory potential and quality with offspring birth outcomes: An individual participant data pooled analysis of 7 European cohorts in the ALPHABET consortium
Source: PLoS Med. 2021 Jan 21;18(1):e1003491. doi: 10.1371/journal.pmed.1003491 (PMC7819611; doi:10.1371/journal.pmed.1003491)
Supplement: S19 Table — (DOCX) [file pmed.1003491.s021.docx]

**S19 Table** Pooled *P*-values for sex-interaction between maternal dietary quality and inflammatory potential and offspring birth outcomes

|  |  |  |  |  |  |  |  |  |  |  |  |  |  |
| --- | --- | --- | --- | --- | --- | --- | --- | --- | --- | --- | --- | --- | --- |
|  | Birthweight, g | Gesta-tional age, wk | Birth length, cm | Head circum-ference, cm | Abdominal circumference, cm | Sum of skinfold thickness, mm |  | Low birth weight | SGA | Macrosomia | LGA | Preterm birth | Post-term birth |
| **E-DII** |  |  |  |  |  |  |  |  |  |  |  |  |  |
| Pre | 0.029 | 0.058 | 0.104 | 0.059 | 0.45 | 0.72 |  | 0.11 | 0.075 | 0.15 | 0.18 | 0.39 | 0.57 |
| Preg | 0.21 | 0.87 | 0.11 | 0.72 | 0.049 | 0.78 |  | 0.54 | 0.33 | 0.21 | 0.12 | 0.39 | 0.81 |
| Early | 0.41 | 0.66 | 0.15 | 0.29 | 0.061 | 0.66 |  | 0.51 | 0.48 | 0.41 | 0.41 | 0.98 | 0.81 |
| Late | 0.086 | 0.044 | 0.46 | 0.62 | 0.63 | 0.68 |  | 0.57 | 0.42 | 0.18 | 0.17 | 0.18 | 0.83 |
|  |  |  |  |  |  |  |  |  |  |  |  |  |  |
| **DASH** |  |  |  |  |  |  |  |  |  |  |  |  |  |
| Pre | 0.13 | 0.23 | 0.71 | 0.62 | 0.62 | 0.86 |  | 0.62 | 0.094 | 0.70 | 0.57 | 0.91 | 0.32 |
| Preg | 0.99 | 0.46 | 0.84 | 0.46 | 0.51 | 0.52 |  | 0.29 | 0.24 | 0.19 | 0.55 | 0.22 | 0.97 |
| Early | 0.70 | 0.27 | 0.28 | 0.77 | 0.44 | 0.41 |  | 0.44 | 0.49 | 0.47 | 0.93 | 0.74 | 0.70 |
| Late | 1.00 | 0.68 | 0.12 | 0.26 | 0.84 | 0.92 |  | 0.73 | 0.35 | 0.23 | 0.40 | 0.64 | 0.52 |

E-DII, energy-adjusted Dietary Inflammatory Index; DASH, Dietary Approaches to Stop Hypertension; SGA, small-for-gestational-age; LGA, large-for-gestational-age; Pre, pre-pregnancy; Preg, pregnancy; Early, early pregnancy; Late, late pregnancy
