# Supplementary material for: Associations of maternal dietary inflammatory potential and quality with offspring birth outcomes: An individual participant data pooled analysis of 7 European cohorts in the ALPHABET consortium
Source: PLoS Med. 2021 Jan 21;18(1):e1003491. doi: 10.1371/journal.pmed.1003491 (PMC7819611; doi:10.1371/journal.pmed.1003491)
Supplement: S20 Table — (DOCX) [file pmed.1003491.s022.docx]

**S20 Table** Sex-interaction between maternal pregnancy E-DII score and offspring birth outcomes (all *P*-interactions ≤0.10)

| Group | Period | Outcome | β (95% CI) |
| --- | --- | --- | --- |
| Male | Pregnancy | Abdominal circumference | -0.04 (-0.17, 0.09) |
| Female | Pregnancy | Abdominal circumference | 0.10 (-0.03, 0.23) |
|  |  |  |  |
| Male | Early pregnancy | Abdominal circumference | -0.05 (-0.25, 0.15) |
| Female | Early pregnancy | Abdominal circumference | 0.07 (-0.06, 0.19) |
|  |  |  |  |
| Male | Late pregnancy | Birth weight | -22.4 (-40.7, -4.2) |
| Female | Late pregnancy | Birth weight | -13.4 (-24.7, -2.2) |
|  |  |  |  |
| Male | Late pregnancy | Gestational age | -0.02 (-0.10, 0.07) |
| Female | Late pregnancy | Gestational age | 0.01 (-0.04, 0.06) |

E-DII, energy-adjusted Dietary Inflammatory Index; LGA, large-for-gestational-age
