# Supplementary material for: Associations of maternal dietary inflammatory potential and quality with offspring birth outcomes: An individual participant data pooled analysis of 7 European cohorts in the ALPHABET consortium
Source: PLoS Med. 2021 Jan 21;18(1):e1003491. doi: 10.1371/journal.pmed.1003491 (PMC7819611; doi:10.1371/journal.pmed.1003491)
Supplement: S21 Table — (DOCX) [file pmed.1003491.s023.docx]

**S21 Table** Association between maternal pregnancy E-DII and DASH scores (per 1-SD increase) and offspring A) continuous outcomes and B) binary outcomes in the Generation R study

A)

|  | Birth weight, g |  | Gestational age,wk |  | Birth length, cm |  | Head circumference, cm |  |
| --- | --- | --- | --- | --- | --- | --- | --- | --- |
|  | β (95% CI) | *n* | β (95% CI) | *n* | β (95% CI) | *n* | β (95% CI) | *n* |
| **E-DII** |  |  |  |  |  |  |  |  |
| W/EB | -36.2 (-53.4, -19.1)*** | 4115 | -0.08 (-0.14, -0.03)** | 4134 | -0.09 (-0.18, 0.001) | 2775 | -0.08 (-0.15, -0.01)* | 2303 |
| NW/NEB | -53.6 (-76.6, -30.7)*** | 2096 | -0.16 (-0.24, -0.09)*** | 2107 | -0.20 (-0.32, -0.07)*** | 1285 | -0.11 (-0.21, -0.01)* | 1103 |
| **DASH** |  |  |  |  |  |  |  |  |
| W/EB | 42.9 (25.4, 60.3)*** | 4115 | 0.09 (0.03, 0.15)** | 4134 | 0.10 (0.01, 0.20)* | 2775 | 0.07 (0.002, 0.14)* | 2303 |
| NW/NEB | 29.3 (4.5, 54.2)* | 2096 | 0.04 (-0.04, 0.12) | 2107 | 0.09 (-0.05, 0.23) | 1285 | 0.04 (-0.06, 0.15) | 1103 |

B)

|  | Low birth weight |  | SGA |  | Macrosomia |  | LGA |  | Preterm birth |  | Post-term birth |  |
| --- | --- | --- | --- | --- | --- | --- | --- | --- | --- | --- | --- | --- |
|  | OR (95% CI) | *n* | OR (95% CI) | *n* | OR (95% CI) | *n* | OR (95% CI) | *n* | OR (95% CI) | *n* | OR (95% CI) | *n* |
| **E-DII** |  |  |  |  |  |  |  |  |  |  |  |  |
| W/EB | 1.21 (1.02, 1.42)* | 4115 | 1.31 (1.12, 1.53)** | 4097 | 0.97 (0.88, 1.06) | 4115 | 0.95 (0.88, 1.03) | 4097 | 1.12 (0.96, 1.30) | 4134 | 0.98 (0.87, 1.11) | 4134 |
| NW/NEB | 1.18 (0.98, 1.43) | 2096 | 1.01 (0.85, 1.20) | 2087 | 0.73 (0.63, 0.85)*** | 2096 | 0.83 (0.73, 0.94) | 2087 | 1.22 (1.01, 1.48)* | 2107 | 0.97 (0.81, 1.17) | 2107 |
| **DASH** |  |  |  |  |  |  |  |  |  |  |  |  |
| W/EB | 0.78 (0.66, 0.93)** | 4115 | 0.69 (0.59, 0.82)*** | 4097 | 1.08 (0.98, 1.18) | 4115 | 1.09 (1.01, 1.19)* | 4097 | 0.88 (0.75, 1.03) | 4134 | 1.03 (0.91, 1.17) | 4134 |
| NW/NEB | 0.95 (0.77, 1.17) | 2096 | 0.91 (0.76, 1.10) | 2096 | 1.19 (1.01, 1.40)* | 2096 | 1.08 (0.95, 1.23) | 2087 | 1.02 (0.83, 1.25) | 2107 | 0.91 (0.74, 1.11) | 2107 |

Values are adjusted pooled effect estimates [β (95% CI)] or [OR (95% CI)] expressed for a 1-SD increment in dietary scores across different outcomes as labelled. Effect estimates were adjusted for maternal education, ethnicity, pre-pregnancy BMI, maternal height, parity, energy intake (for DASH), cigarette smoking and alcohol consumption during pregnancy, and child sex.

E-DII, energy-adjusted Dietary Inflammatory Index; DASH, Dietary Approaches to Stop Hypertension; W/EB: White/European-born; NW/NEB: non-White/non-European-born; SGA, small for gestational age; LGA, large for gestational age
